# Genesis of Mammalian Prions: From Non-infectious Amyloid Fibrils to a Transmissible Prion Disease

**DOI:** 10.1371/journal.ppat.1002419

**Published:** 2011-12-01

**Authors:** Natallia Makarava, Gabor G. Kovacs, Regina Savtchenko, Irina Alexeeva, Herbert Budka, Robert G. Rohwer, Ilia V. Baskakov

**Affiliations:** 1 Center for Biomedical Engineering and Technology, University of Maryland, Baltimore, Maryland, United States of America; 2 Institute of Neurology, Medical University of Vienna, Vienna, Austria; 3 Medical Research Service, Veterans Affairs Medical Center, University of Maryland, Baltimore, Maryland, United States of America; 4 Department of Anatomy and Neurobiology, University of Maryland, Baltimore, Maryland, United States of America; Dartmouth Medical School, United States of America

## Abstract

The transmissible agent of prion disease consists of a prion protein in its abnormal, β-sheet rich state (PrP^Sc^), which is capable of replicating itself according to the template-assisted mechanism. This mechanism postulates that the folding pattern of a newly recruited polypeptide chain accurately reproduces that of a PrP^Sc^ template. Here we report that authentic PrP^Sc^ and transmissible prion disease can be generated *de novo* in wild type animals by recombinant PrP (rPrP) amyloid fibrils, which are structurally different from PrP^Sc^ and lack any detectable PrP^Sc^ particles. When induced by rPrP fibrils, a long silent stage that involved two serial passages preceded development of the clinical disease. Once emerged, the prion disease was characterized by unique clinical, neuropathological, and biochemical features. The long silent stage to the disease was accompanied by significant transformation in neuropathological properties and biochemical features of the proteinase K-resistant PrP material (PrPres) before authentic PrP^Sc^ evolved. The current work illustrates that transmissible prion diseases can be induced by PrP structures different from that of authentic PrP^Sc^ and suggests that a new mechanism different from the classical templating exists. This new mechanism designated as “deformed templating” postulates that a change in the PrP folding pattern from the one present in rPrP fibrils to an alternative specific for PrP^Sc^ can occur. The current work provides important new insight into the mechanisms underlying genesis of the transmissible protein states and has numerous implications for understanding the etiology of neurodegenerative diseases.

## Author Summary

The transmissible agent of prion disease consists of a prion protein in its abnormal conformation (PrP^Sc^), which replicates itself according to the template-assisted mechanism. This mechanism postulates that the folding pattern of a newly recruited polypeptide chain accurately reproduces that of a PrP^Sc^. The current study reports that infectious prions and transmissible prion disease can be triggered in wild type animals by amyloid fibrils produced from recombinant prion prtotein, which are structurally different from PrP^Sc^ and lacks any detectable PrP^Sc^ particles. This work introduces a new hypothesis that transmissible prion diseases can be induced by prion protein structures different from that of authentic PrP^Sc^ and suggests that a new mechanism for triggering PrP^Sc^ formation different from the classical templating exists. The current work provides important new insight into the mechanisms underlying genesis and evolution of the transmissible states of the prion protein and has numerous implications for understanding the etiology of prion and other neurodegenerative diseases.

## Introduction

Prion diseases, or transmissible spongiform encephalopathies (TSEs), are fatal neurodegenerative disorders that can be sporadic, inherited, or infectious in origin. Misfolding and aggregation of the normal, cellular form of the prion protein (PrP^C^) into an abnormal β-sheet rich state underlies the pathogenic mechanisms of the prion diseases for all three origins [1]. The “protein only” hypothesis of prion propagation postulates that the transmissible agent of prion diseases consists of a prion protein in its abnormal, β-sheet rich conformation (PrP^Sc^), which is capable of propagating itself in an autocatalytic manner by recruiting and converting PrP^C^ [2], [3].

Recent years have witnessed a number of studies where transmissible prion diseases were generated in animals *de novo* by inoculating prion infectious material produced *in vitro* [4]–[10]. All studies on generating prion infectivity can be divided into two large groups, where the materials for inoculating animals were produced using either (i) Protein Misfolding Cyclic Amplification (PMCA) [8]–[10], or (ii) *in vitro* fibrillation protocols that utilized recombinant PrP (rPrP) [4]–[7]. In the studies that employed the first approach, application of serial PMCA (sPMCA) accomplished two purposes: (i) generating PrP^Sc^ particles *de novo* and (ii) amplification of newly formed PrP^Sc^ to amounts that can effectively produce clinical disease in wild type animals within a short incubation time and 100% success rate [8]–[10]. Because *de novo* generation of PrP^Sc^ in sPMCA followed stochastic behavior, it remains unclear whether a large amount of infectivity produced after multiple rounds of sPMCA was a result of amplification of a few or even a single PrP^Sc^ particle. In the second approach, rPrP was converted into amyloid fibrils *in vitro* without application of PMCA [4]–[7]. In these studies, transmissible diseases were generated either in transgenic animals with high levels of PrP^C^ expression and after relatively long incubation time [4], [5], [7], or in wild type animals, but with a less than 100% success rate and after a long silent stage [6]. Critical concerns that rPrP amyloid fibrils did not induce the disease *de novo* but only accelerated an on-going pathogenic process have been raised regarding the studies performed on transgenic mice [11]–[13]. For the experiments conducted on wild type animals, a long silent stage and less than 100% success rate led to the general assumption that the preparations of amyloid fibrils contain minuscule amounts of PrP^Sc^ or particles with a structure of PrP^Sc^, and that this tiny sub-fraction is responsible for the disease.

In the current study we report that no PrP^Sc^ can be detected in preparations of rPrP amyloid fibrils using a sPMCA format that could detect as little as a single PrP^Sc^ particle. Nevertheless, despite lack of PrP^Sc^ in the preparations of rPrP fibrils, transmissible prion disease was produced in wild type animals. When induced by rPrP fibrils, which are known to be structurally different from PrP^Sc^ [14]–[17], the clinical disease developed only at the third serial passage. The long silent stage to the disease that involved two serial passages was found to be accompanied by significant changes in biochemical and neuropathological properties of the proteinase K (PK) -resistant PrP (PrPres). These changes involved dramatic transformation of a PK-resistant profile, changes in the PrPres deposition pattern and expansion of brain areas targeted by PrPres. Clinical, neuropathological, and biochemical features of the new prion disease were different from those produced in rodents by previously known, hamster-adapted prion strains or strains generated by sPMCA [8]–[10], consistent with the idea that the new strain originated from a unique amyloid structure. The current work introduces a new hypothesis that transmissible prion diseases can be induced by cross-β PrP structures substantially different from that of authentic PrP^Sc^. This hypothesis suggests that a new templating mechanism (which will be referred to as “deformed templating”) different from the classical templating might exist. The deformed templating involves a switch from one PrP folding pattern present in amyloid fibrils to an alternative folding pattern present in PrP^Sc^. The current work provides important new insight into the mechanisms underlying genesis and evolution of the transmissible states of the prion protein and has numerous implications for understanding the etiology of prion and other neurodegenerative diseases.

## Results

### Serial transmission of rPrP amyloid fibrils

**Figure 1:**
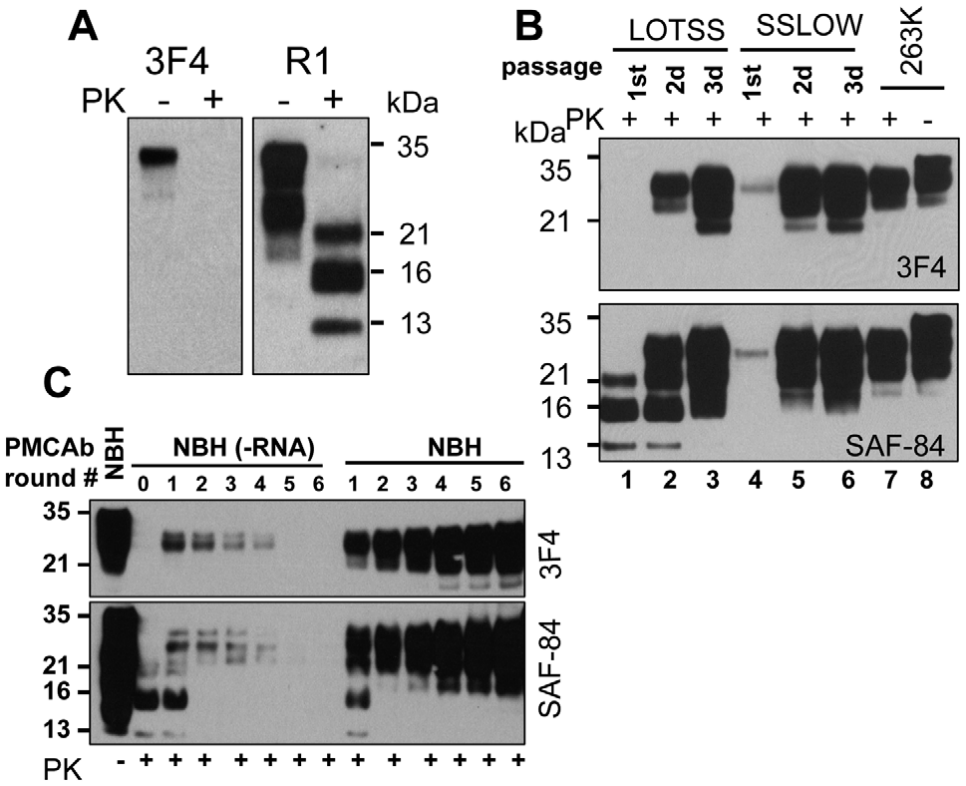
Serial passages of rPrP fibrils and *de novo* generation of PrP^Sc^ in Syrian hamsters. (**A**) Western blotting of brain homogenate (BH) from the animal inoculated with BSA-annealed rPrP amyloid fibrils. Western blots were stained with 3F4 (left panel) or R1 (right panel) antibody. Undigested samples were loaded at 1/10^th^ the amount of the digested samples. (**B**) Western blotting of BHs of animals from the 1^st^, 2^nd^, or 3^rd^ passages of BSA-annealed rPrP fibrils (lanes 1, 2 and 3, respectively) stained with 3F4 (top panel) or SAF-84 (bottom panel) antibody. The new prion strain produced as a result of inoculation of BSA-annealed rPrP fibrils will be designated as LOTSS. The 1^st^, 2^nd^, or 3^rd^ passages of SSLOW, the strain that was previously produced as a result of inoculation of normal BH (NBH)-annealed rPrP fibrils (lanes 4, 5 and 6 respectively) [6], or 263 K are provided as references. (**C**) sPMCAb of brain material from the 1^st^ passage of LOTSS. 10% BH from the animal inoculated with BSA-annealed rPrP amyloid fibrils was diluted 10-fold into 10% RNA-depleted (NBH(-RNA)) or NBH and subjected to sPMCAb. Each PMCAb round consisted of 48 cycles, 30 min each; 10-fold dilutions were used for each subsequent sPMCAb round. Western blots were stained with 3F4 (top panel) or SAF-84 (bottom panel) antibody. Undigested 10% NBH is provided as a reference.

Full-length Syrian hamster rPrP was converted into amyloid fibrils *in vitro* and subjected to an annealing procedure (brief heating to 80°C) in the presence of BSA prior to inoculation as previously described [6], [18]. All experiments on expression and purification of rPrP, its conversion into fibrils, annealing and preparation of inocula were performed in a facility and with equipment that have never been exposed to TSEs. Annealed rPrP fibrils were inoculated intracerebrally into Syrian hamsters. While none of the animals developed any signs of prion disease for up to 661 days post inoculation, one out of seven animals showed atypical, PK-resistant, low molecular weight (LMW) bands of 21, 16 and 13 kDa ([6] and Figure 1A). These PK-resistant material (PrPres) could be detected by the antibodies against the C-terminal PrP region R1 (epitope 225–231) or SAF-84 (epitope 160–170), but not by 3F4 (epitope 109–112) (Figure 1A and B). Interestingly, when used as seed in sPMCA reaction with beads (sPMCAb) [19] , the brain material with atypical PrPres gave rise to PK-resistant products with a band-shift typical for authentic PrP^Sc^ that could be detected by 3F4 and the C-terminal antibodies (Figure 1C). These PK-resistant products will be referred to as typical or standard PrPres. We do not know whether it was atypical PrPres that gave rise to typical PrPres in sPMCAb or small, undetectable amounts of typical PrPres that might have already been present in brain homogenates (BH).

BHs from the remaining six animals that lacked any PrPres material on Western blot were tested in PMCAb. After a single PMCAb round, BH from one more animal showed PrPres products detectible by 3F4 antibody (Figure S1A). By the sixth sPMCAb round, BH from an additional animal showed PrPres positive by 3F4 antibody (Figure S1B). Ten non-seeded sPMCAb reactions were all negative after six sPMCAb rounds (Figure S1C) arguing against possibility of cross-contamination or generation of PrP^Sc^ or PrPres *de novo* in the sPMCAb format employed here. As we reported previously, sPMCAb reactions seeded with normal BHs (NBHs) from old animals were also all negative after six sPMCAb rounds [19]. Therefore, three out of seven animals inoculated with rPrP fibrils showed signs of infection as judged by appearance of PMCAb-active PrPres material in their brains, although the amounts of PrPres material was highly variable. As previously described, no clinical signs or PrPres material were found in animals inoculated with any of the controls or in uninoculated age-matched controls ([6] and Table S1).

To perform a second passage, 10% BH containing atypical PrPres was inoculated into a new group. Surprisingly, none of the animals developed clinical signs of the disease before they were euthanized at 661 days (Table S1). However, six out of seven hamsters showed large amounts of standard and atypical PrPres (Figures 1B and S2). The remaining animal showed predominantly atypical, LMW PrPres detectible by SAF-84 antibody and miniscule amounts of standard PrPres (Figure S2, lane 6). After a single PMCAb round, BH from this animal showed PrPres products detectible by 3F4 antibody (Figure S2, lane 9). These results illustrated that all animals were infected in a second passage. The variation in the ratio of standard to atypical PrPres in animals from the 2^nd^ passage (Figure S2) suggested that transformation in PrPres structure and PK-resistance pattern occurred at a variable rate within the same group of animals. Considering that no clinical signs were observed despite substantial amounts of PrPres, the new synthetic strain was designated as LOTSS (LOw Toxicity Synthetic Strain).

The amount of PrPres in LOTSS-infected animals from the 2^nd^ passage was similar to those found at the terminal stage of 263 K-inoculated hamsters (Figure 1B). While no signs of clinical disease were observed, LOTSS PrPres from the 2^nd^ passage showed biochemical attributes of authentic PrP^Sc^. LOTSS standard PrPres could be amplified in sPMCAb and displayed an amplification rate similar to that of 263 K PrP^Sc^ (Figure S3A). The resistance of LOTSS standard PrPres to PK digestion was similar to that of 263 K (Figure S3B).

**Figure 2:**
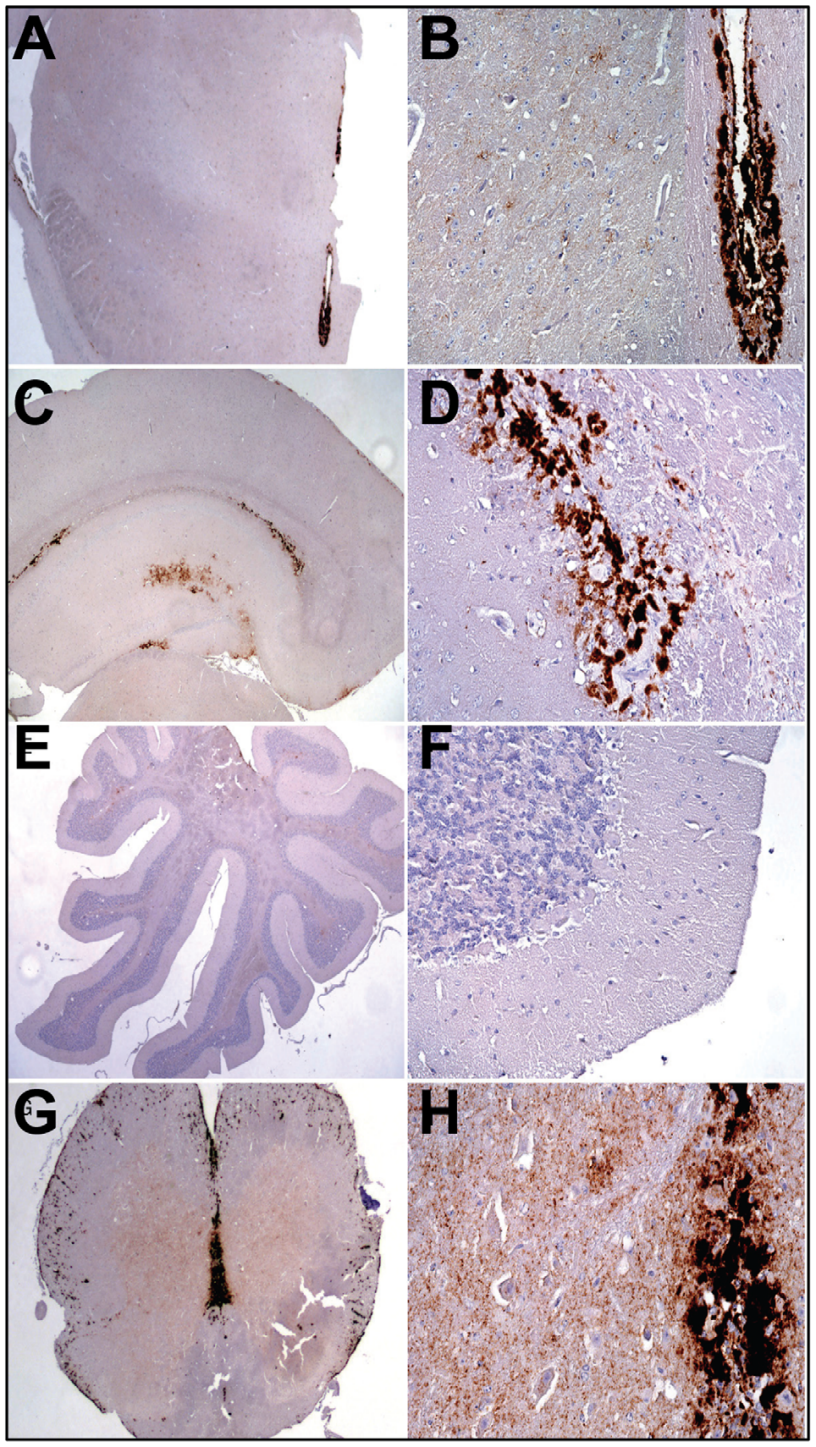
Analysis of PrP deposition in LOTSS-inoculated animals from the 2d passage. (**A, B**) PrP plaques in the subependymal regions and minor synaptic immunoreactivity in the basal ganglia and thalamus. (**C, D**) PrP plaques in the subependymal regions in hippocampus and frontal cortex. (**E, F**) Very minor focal PrP deposition in cerebellum. (**G, H**) Plaques around the aqueductus and synaptic PrP immunoreactivity in the posterior and anterior horns of spinal cord.

**Figure 3:**
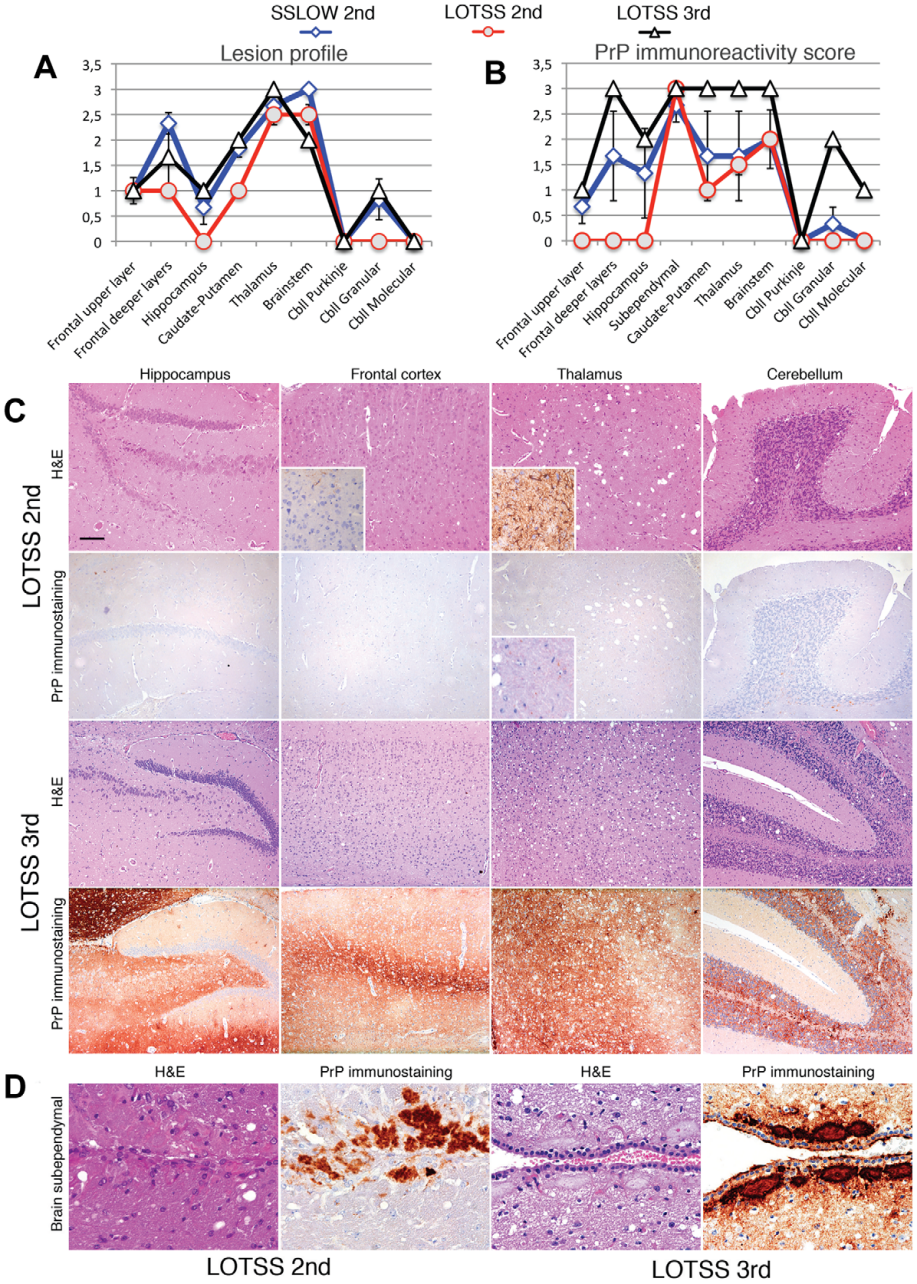
Histopathological analysis of brains of LOTSS-inoculated animals. Lesion profile (**A**) and PrP immunopositivity score (**B**) for hamsters from the 2^nd^ (o, red lines) or 3^rd^ passages (Δ, black lines) of LOTSS. The histopathological profiles for SSLOW-inoculated hamsters (2^nd^ passage, ◊ blue line) were previously described in [6] and are provided as a reference. The lesion profile was obtained by averaging the scores for spongiform change, neuronal loss and gliosis for three animals within each group. (**C**) Comparison of spongiform changes in the hippocampus, frontal cortex, thalamus, and cerebellum stained with Hematoxylin and Eosin (H&E; upper panels) or anti-PrP 3F4 antibody (lower panels) in hamsters from 2^nd^ or 3^rd^ passages of LOTSS, as indicated. Insets in H&E stained images of the frontal cortex and thalamus from LOTSS 2^nd^ animals represent GFAP immunostaining, and enlargement of the PrP immunostaining. (**D**) Brain subependymal region stained with H&E and 3F4 antibody in LOTSS-inoculated animals from 2^nd^ (left panel) or 3^rd^ (right panel) passages. Scale bar = 00 µm in **C** and = 60 µm in **D**.

Histopathological studies of animals from the 2^d^ passage of LOTSS revealed characteristic signs of TSE infection including spongiform degeneration, neuronal loss, reactive astrogliosis and deposition of disease-associated PrP in the brains and spinal cords (Figures 2 and 3). No inflammatory changes were observed in the brains and no immunoreactivity for disease-associated PrP was found in the spleens (data not shown). Spongiform change, neuronal loss, and reactive astrogliosis were predominantly found in the thalamus and the brainstem followed by caudate-putamen and spinal cord, however, they were minor in the frontal cortex, hippocampus or cerebellum (Figure 3A and C). Similar to the animals infected with SSLOW, a synthetic strain that was previously produced as a result of inoculation of NBH-annealed rPrP fibrils [6], LOTSS-infected brains from the 2^nd^ passage displayed prominent, large plaques and amorphous deposits in the subpial, periventricular and periaqueductal subependymal regions (Figures 2 and 3). Only mild synaptic PrP immunoreactivity was found in the thalamus, brainstem, caudate-putamen and spinal cord, while no noticeable synaptic PrP deposition was observed in frontal cortex, hippocampus, or cerebellum (Figures 2 and 3C). Previously, synaptic PrP immunoreactivity was defined as a type of disease-associated PrP that co-localizes with synaptophysin [20]. Overall, the lesion profile and the pattern of PrP immunostaining was reminiscent of those observed in SSLOW-inoculated animals [6], but significantly different from those in 263 K-infected brains.

### Differences between LOTSS and SSLOW

**Figure 4:**
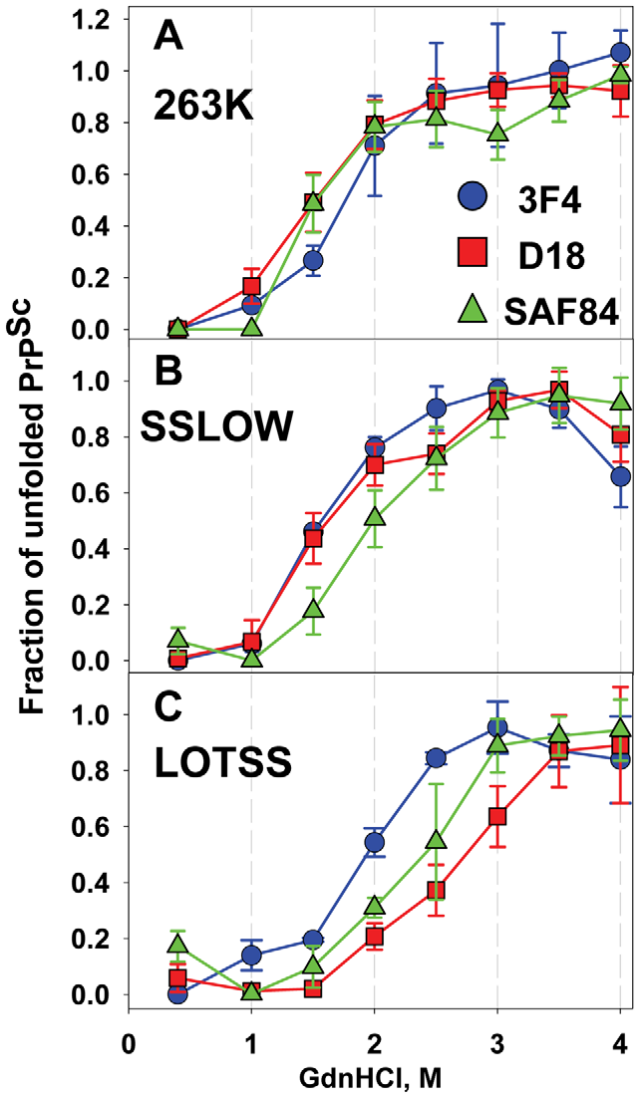
Analysis of epitope-specific conformational stability. Conformational stability profiles for 263 K (A), SSLOW (B), or LOTSS (C) monitored in GdnHCl-induced denaturation assay using 3F4 (•, blue), D18 (▪, red) or SAF-84 (▴, green) antibodies. Data represent average ± SD of four denaturation experiments. Brain materials from SSLOW- or LOTSS-inoculated animals from the 2^nd^ passages were used. 96-well dot blot was used instead of Western blot for quantitative analysis of multiple replicas in a single blot assay.

In previous studies, we showed that serial passaging of rPrP fibrils annealed in NBH in Syrian hamster produced a synthetic strain (designated as SSLOW) with highly unique clinical and neuropathological features [6]. To test whether LOTSS is different from SSLOW or 263 K, we employed a conformational stability assay that monitors GdnHCl-induced denaturation using 3F4, D18 and SAF-84 antibodies that recognize PrP regions 109–112, 133–157 and 160–170, respectively (Figure 4). Consistent with previous study [21], the denaturation profiles monitored by three antibodies were superimposable for 263 K (Figure 4A). This result suggests that 263 K follows apparent two-state unfolding without notable stable intermediates. In contrast to 263 K, SSLOW showed a dissociation of the denaturation profile monitored by SAF-84 from those monitored by 3F4 or D18 (Figure 4B), while LOTSS exhibited dissociation of the profiles monitored by D18 and SAF-84 from that of 3F4 (Figure 4C). This experiment revealed that (i) three strains showed three individual strain-specific unfolding patterns; (ii) SSLOW and LOTTS exhibited more complex unfolding behavior than 263 K, (iii) in LOTSS, the central and the C-terminal domain were found to be more stable than the N-terminal domain. Furthermore, the region tracked by D18 antibody was found to be considerably more stable in LOTSS than in SSLOW or 263 K. An alternative experimental format of the conformational stability assay that employs PK-digestion and Western blot instead of dot blot confirmed that three strains were conformationally different (Figure S4).

In addition to the strain-specific differences in unfolding pattern, LOTSS was found to be different from SSLOW or 263 K with respect to the ratio of intensities of SAF-84 to 3F4 immunostaining. Regardless of whether BHs were treated with PK or not and regardless of whether Western blot or dot were used for analysis, LOTSS-infected BHs consistently showed substantially higher ratios of SAF-84 to 3F4 intensities than SSLOW or 263 K (Figures S5 and 1B).

### RNA-dependency emerged during the first passage

Previous studies established that RNA serves as a cofactor in replication of hamster prion strains [22], [23]. Furthermore, formation of authentic mammalian PrP^Sc^ *in vitro de novo* from purified PrP^C^ or rPrP was also shown to require RNA [9], [10]. Because the current study employed the fibrillation protocols that lacked RNA or any other cellular components, rPrP amyloid fibrils did not require RNA for forming or self-propagating their individual cross-β structures [18]. Therefore, we were interested in testing whether RNA-dependency evolved with a transformation of rPrP amyloid structure into a structure of PrPres. To address this question, sPMCAb reactions were seeded with BH that contained atypical PKres products from the 1^st^ passage, and amplification was carried out using normal or RNA-depleted BHs as a source of PrP^C^. In the presence of RNA, a gradual increase in the amounts of standard, 3F4-positive PrPres material was observed with each subsequent round (Figure 1C). This result illustrates that in the presence of RNA, LOTSS PrPres amplification rate exceeded 10-fold per round. However, in RNA-depleted BH, the amounts of PrPres decreased with each subsequent round and disappeared below detectable levels by the 5^th^ round suggesting that the amplification rate was less than 10-fold per round (Figure 1C). In our experience, this amplification rate was similar to the rate displayed by 263 K in RNA-depleted NBH (data not shown). Therefore, in contrast to amyloid fibrillation reactions, the amplification of LOTSS PrPres showed a strong RNA-dependency, just like amplification of other hamster strains [23]. Notably, an increase in PK-resistant material during PMCAb was entirely attributed to the amplification of standard PrPres, whereas no detectible amplification of the atypical PrPres that produces 13- or 16-kDa PK-resistant bands was observed regardless of the presence or absence of RNA. Overall, this experiment revealed that the transformation from the RNA-independent structure of amyloid fibrils to an RNA-dependent structure of LOTSS PrPres occurred during the first passage.

### Evolution of LOTSS neuropathological and biochemical features during serial transmission

Despite substantial amounts of PrPres in the brains and the fact that LOTSS PrPres exhibits key physical attributes of authentic PrP^Sc^, LOTSS-infected animals remained healthy in the second passage. There is a possibility that the clinical signs associated with LOTSS were not clearly visible and only involved impairment of cognitive functions. Lack of clinical signs typical for TSEs might indicate that LOTSS is an intrinsically silent strain, i.e. it is characterized by transmissible formation of PrPres that displays minimal neurotoxicity. Alternatively, LOTSS might not be silent but the second passage represents a silent stage in evolution of LOTSS pathological features. To distinguish between these alternatives, a third passage was conducted. The very first clinical signs of the disease including startle response to sound and touch were noticed starting from 10^th^ to 12^th^ months post inoculation. Similarly to SSLOW-inoculated animals, the progression of disease in LOTSS-infected animals was very slow. After the first signs, 5 to 8 months were required for the disease to progress to the terminal stage (Table S1). Overall, the set of clinical symptoms was similar to that of SSLOW strains (severe obesity, dry hair and hair loss, difficulty in righting, substantially reduced activity) but different from previously described hamster strains.

**Figure 5:**
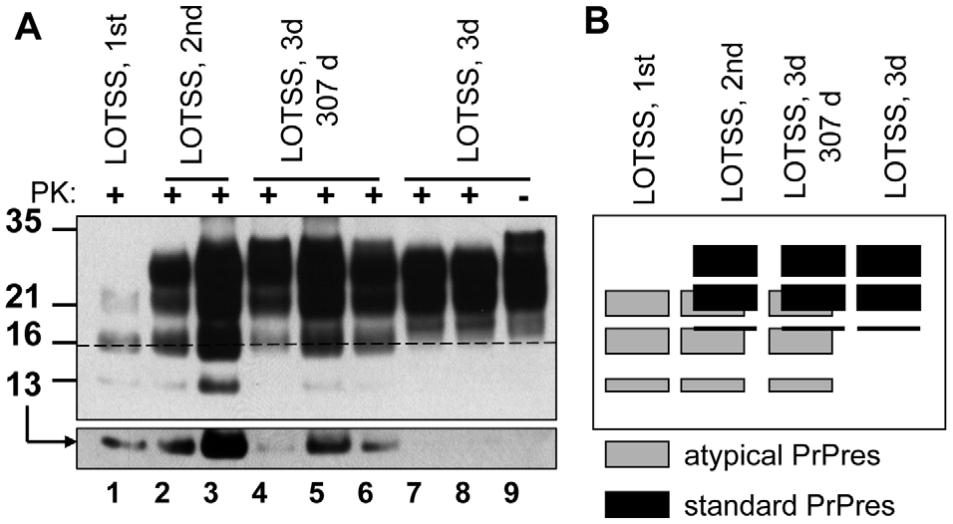
Evolution of PrPres during LOTSS serial transmission. (**A**) Western blotting of BHs of animals from the 1^st^, 2^nd^, or 3^rd^ passages of LOTSS stained with SAF-84 antibodies. Animals from the 1^st^ and 2^nd^ were euthanized at 661 days postinoculation in the absence of clinical disease, whereas animals from the 3^rd^ passage were euthanized at the beginning of first clinical signs, i.e. 307 days postinoculation (lanes 4, 5 and 6), or at the end of the terminal stage (lanes 7, 8). Overexposed Western blot with 13 kDa atypical PrPres fragment is shown in bottom panel. Dashed line marks the central position of the atypical 16 kDa band. Undigested 10% NBH is provided as a reference. (**B**) Schematic representation of the dynamics in PrPres profile during serial transmission, where gray boxes represent atypical PrPres, whereas black boxes represent standard PrPres.

Three animals were analyzed at 307 days postinoculation, at which two animals were yet asymptomatic, while the third one began to show the first signs. While the PrPres amount varied between these three animals, with the largest amount observed in the symptomatic brain, the relative ratio of atypical PrPres (positive for SAF-84) to standard PrPres (positive for 3F4 and SAF-84) remained approximately the same and was similar to those observed in majority of animals at the end of the 2^nd^ passage (Figure 5A). Because of the complexity in the PKres profile and, specifically, the overlap between the upper 21 kDa band of atypical PrPres with the middle band of standard PrPres, the dynamics in PrPres profile is shown in parallel as a schematic diagram (Figure 5B). The variation in the total PrPres amounts by 307 days was similar to the range seen at the end of life on the 2^nd^ passage (Figure 5A).

The remaining animals of the 3^rd^ passage were euthanized between 485 and 568 days postinoculation, as they approached the terminal stage (Table S1). Only a very modest if any increase in the total amount of PrPres was observed during the progression of clinical disease (Figure 5A). Surprisingly, the relative ratio of atypical to standard PrPres changed dramatically within the clinical stage (Figure 5A). As judged from the disappearance of the 16 and 13 kDa bands, atypical PrPres was fully replaced by standard PrPres as the clinical disease progressed to the terminal stage.

Overall, the atypical PrPres was observed during three serial passages, however, its amount diminished to undetectable levels during the 3^rd^ passage clinical stage (Figures 1B and 5). While the atypical PrPres was capable of replicating in a brain, this isoform was not amplifiable under standard sPMCAb conditions (Figure 1C). This first signs of standard PrPres appeared in the first passage, although at the levels detectible only by PMCAb, but not Western blotting (Figure 1A and C). While the accumulation rate of standard PrPres varied between animals within the same group (Figure S2), the amount of standard PrPres continued to increase gradually during the 2^nd^ and 3^rd^ passages until it fully replaced the atypical PrPres by the end of the 3^rd^ passage.

**Figure 6:**
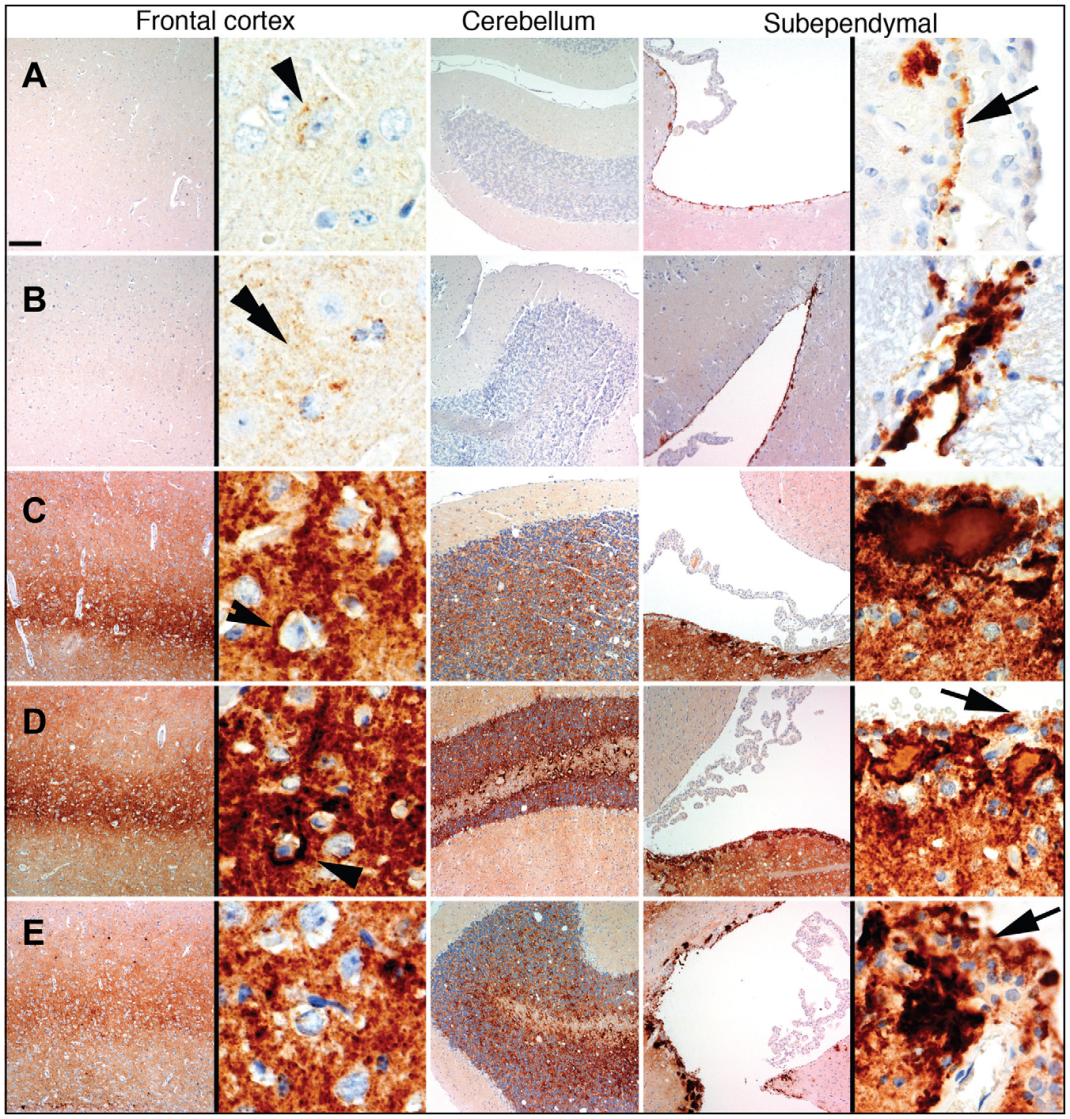
Comparison of PrP deposition at the early and late stages of the disease during the 3^rd^ passage. PrP deposition in frontal cortex, cerebellum or subependymal areas in two animals euthanized at 307 days postinoculation (**A, B**), and three animals euthanized at the terminal stage of the disease at 494, 521, and 494 days postinoculation (**C**, **D**, and **E**, respectively). Scale bar upper left represents 40 µm for all except for the right panels of the frontal cortex and subependymal images where it represents 5 µm. Arrowheads indicate perineuronal deposits, double arrowhead indicates fine synaptic immunoreactivity and arrows indicate granular immunoreactivity around ependymal cells.

Neuropathological analysis of animals euthanized at 307 days postinoculation in a 3^rd^ passage revealed plaques and granular PrP deposits in the subpial, periventricular, and subependymal areas, as well as fine perineuronal and synaptic PrP immunoreactivity in thalamus and basal ganglia (Figure 6). These features were similar to those observed in LOTSS-inoculated animals from the 2^nd^ passage (Figure 2). However, in contrast to the animals from the 2^nd^ passage, the frontal cortex showed prominent diffuse synaptic PrP immunoreactivity and perineuronal PrP deposition (Figure 6).

Upon development of clinical disease in animals from the 3^rd^ passage, a dramatic increase in synaptic PrP immunoreactivity was observed in all examined regions, including the hippocampus, frontal cortex, thalamus, and granular layer of the cerebellum (Figures 3C and 6). In the animals from the 2^nd^ LOTSS passage, only a few brain regions showed weak synaptic PrP immunoreactivity indicating that this pathological hallmark had been evolving with serial transmission (Figures 2 and 4C). Furthermore, the progression of clinical disease was accompanied by accumulation of prominent perineuronal PrP deposition in the deeper layers of the frontal cortex (Figure 6) and thalamus. During the clinical course, the smaller plaques or granular deposits in the subependymal areas evolved into large plaques (Figure 6). In addition, at the terminal stage, perivascular miniplaques appeared in the cerebellum and brainstem (Figure 6 C, D and E).

Overall, LOTSS 3^rd^ passage presented with a quantitative and qualitative change in the neuropathological phenotype. While subependymal plaques were observed in animals from both 2^nd^ and 3^rd^ passages, the animals of the 3^rd^ passage were characterized by substantially more pronounced PrP immunoreactivity including immunopositivity in the regions that did not show any PrP deposition in LOTSS 2^nd^. In addition, prominent diffuse synaptic and perineuronal PrP deposition appeared as a correlate for the clinical symptoms in LOTSS 3^rd^ indicating that the lack of clinical disease in 2^nd^ passage could be attributed to the limited synaptic deposition. These results suggest that LOTSS pathological features evolve over substantial time period, which might take several serial passages. Comparisons of PrP immunoreactivity score shows that new brain regions including frontal layers, hippocampus and cerebellum became involved during the 3^rd^ passage (Figure 3B). The lesion profile obtained by averaging the spongiform changes, neuronal loss and gliosis was found to be very similar in LOTSS- and SSLOW-inoculated animals but different from the profiles in other hamster strains suggesting that LOTSS and SSLOW belong to the same family of strains (Figure 3A).

### Preparation of rPrP amyloid fibrils lacks any detectible PrP^Sc^

Two alternative mechanisms can be put forward to explain the low success rate in triggering transmissible prion disease by rPrP amyloid fibrils. According to one mechanism, the preparations of rPrP amyloid fibrils contain very small amounts of PrP^Sc^ or particles with the structure of authentic PrP^Sc^. If this is true, the low success rate and long silent stage of the disease are attributed to the long time required for amplification of this extremely small amount of PrP^Sc^. An alternative mechanism postulates that there are no PrP^Sc^ particles in the preparations of amyloid fibrils. Instead, amyloid fibrils, while structurally different from PrP^Sc^ [14]–[17], nevertheless are capable of triggering formation of PrP^Sc^ when inoculated into animals. In this case, the low success rate and silent stage of the disease are attributed to inefficient seeding of PrPres by recombinant PrP amyloids and subsequent evolution of PrPres structural and biological features, a process that eventually give rise to PrP^Sc^.

**Figure 7:**
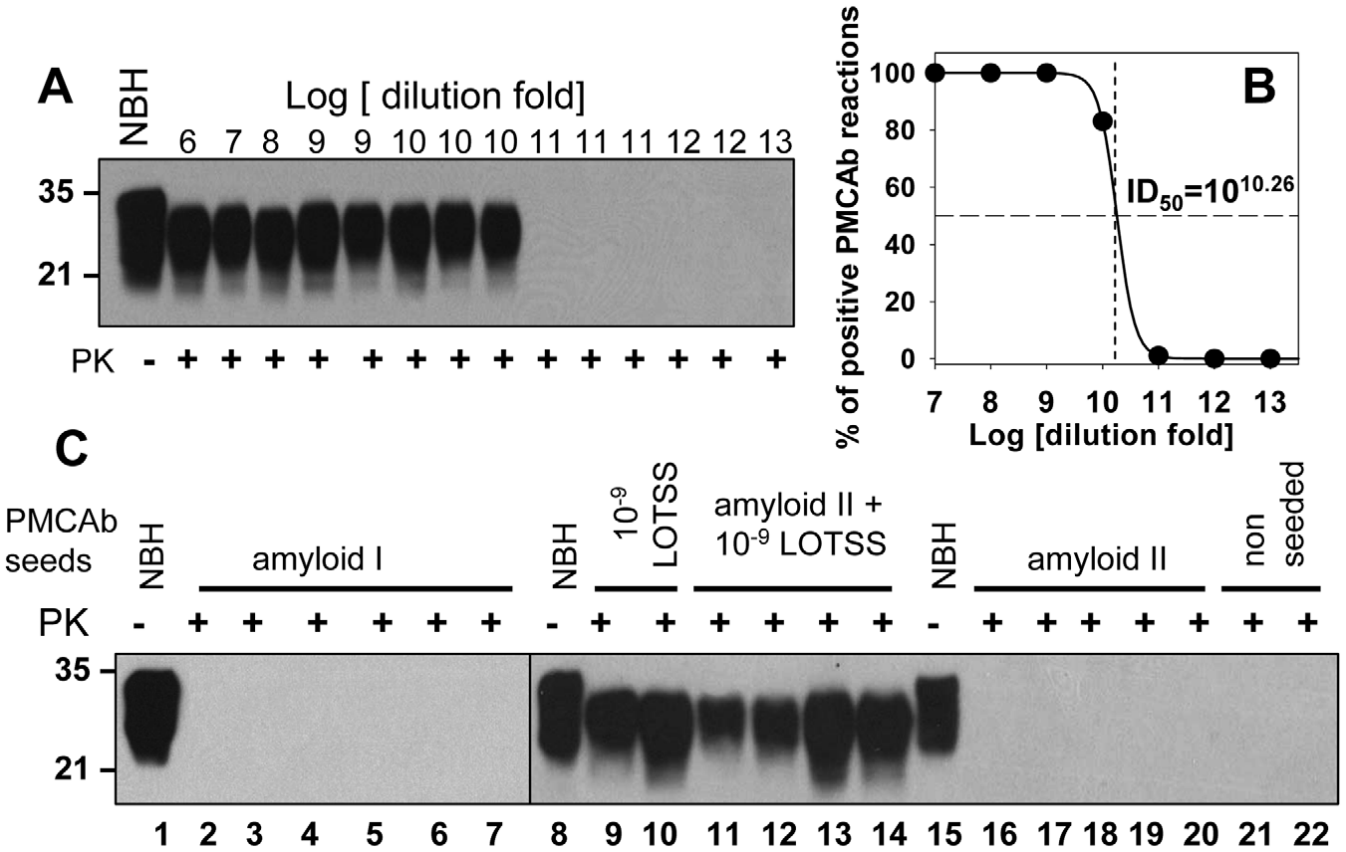
Preparations of rPrP fibrils have no detectible PrP^Sc^. PMCAb titration of LOTSS brain material **(A** and **B**). (**A**) LOTSS brain material was serially diluted to up to 10^13^-fold and each dilution was subjected to six rounds of sPMCAb. Representative results are shown on Western blot stained with 3F4 antibody. Undigested 10% NBH is provided as a reference. (**B**) For each dilution of brain material, the fractions of sPMCAb reactions with a positive signal on Western blot were plotted against the logarithm of dilution (•) and non-linear least-square regression to a sigmoidal function (black solid line, R = 0.9999) was used to calculate PMCAb_50_ titer. (**C**) sPMCAb reactions were seeded with two independent preparations of BSA-annealed rPrP amyloid fibrils (lanes 2–7 with amyloid I or lanes 16–20 with amyloid II), 10^9^-fold diluted LOTSS brain material (lanes 9, 10), or with amyloid fibrils II and 10^9^-fold diluted LOTSS brain material (lanes 11–14), then six rounds of sPMCAb were conducted for each condition. The final concentraton of rPrP amyloid fibrils in sPMCAb reaction was 10 µg/ml for amyloid I or 5 µg/ml for amyloid II. Two non-seeded reactions are shown as negative control. Brains from LOTSS-inoculated animals from the 2^nd^ passages were used for all experiments. Undigested 10% NBH is provided as a reference. Western blots were stained with 3F4.

To distinguish between two mechanisms, we employed sPMCAb that can detect miniscule amounts of PrP^Sc^. In the first series of experiments, we tested whether miniscule amounts of LOTSS PrP^Sc^ could be efficiently detected by sPMCAb. 10% BHs prepared from the 2^nd^ passage of LOTSS were diluted in ten fold serial steps, then aliquots from each dilution were used to seed sPMCAb reactions (Figure 7A). In 100 µl of PMCAb reaction volume, 10^9^- and 10^10^-fold diluted LOTSS brain material was detected with 100% and 83% success rate, respectively (Figure 7B). The reactions seeded with brain material diluted to 10^11^-fold or higher were all negative. Six sPMCAb rounds were sufficient for amplification of the highest dilutions of LOTSS brain material to the level detectible by Western blot. Non-seeded normal BH subjected to six sPMCAb rounds were all negative illustrating that positive signals were not due to spontaneous formation or cross-contamination (Figure S1C). As judged from the fitting of experimental data on sPMCAb amplification of serially diluted LOTSS brain material (Figure 7B), a PMCAb_50_ titer was found to be 10^10.26^ (Figure 7B). This value represents a dilution fold at which there is a 50% chance of finding at least one PMCAb-active PrP^Sc^ particle capable of initiating PMCAb amplification per 100 µl of the reaction volume. This experiment demonstrated that the sPMCAb format employed here can effectively detect miniscule amounts and, presumably, as little as a single LOTSS PrP^Sc^ particle.

In the next series of experiments, sPMCAb was employed for detecting PrP^Sc^ in the preparations of rPrP amyloid fibrils subjected to annealing in the presence of BSA. Eleven sPMCAb reactions seeded with rPrP amyloid fibrils from two independent amyloid preparations were conducted. No positive signals were detected in any reactions after six sPMCAb rounds (Figure 7C). As judged from the previous experiment on PMCAb titration, six sPMCAb rounds were sufficient for detecting LOTSS PrP^Sc^ at the level of a single particle in 100 µl of the reaction volume.

To rule out the possibility that the negative result in detecting PrP^Sc^ in the preparations of amyloid fibrils was due to the inhibitory effect of fibrils on amplification of PrP^Sc^, sPMCAb reactions were seeded with rPrP amyloid fibrils mixed with 10^9^-fold diluted LOTSS brain material. 10^9^-fold diluted brain material represents the highest dilution at which 100% sPMCAb reactions were positive in the PMCAb titration experiment. Four independent sPMCAb reactions were conducted and all gave a positive signal on Western blot (Figure 7C). This experiment confirmed that the presence of rPrP fibrils did not diminish the sensitivity of detection of LOTSS PrP^Sc^ by sPMCAb. Taken together, these experiments revealed that the preparations of rPrP amyloid fibrils do not contain any PrP^Sc^ particles that could be detected by sPMCAb providing strong support for the second mechanism. In our experience and consistent with the previously published data [24], sPMCAb is ∼400 to 4000 fold more sensitive than bioassay.

### Transition from rPrP fibrils to LOTSS PrP^Sc^

**Figure 8:**
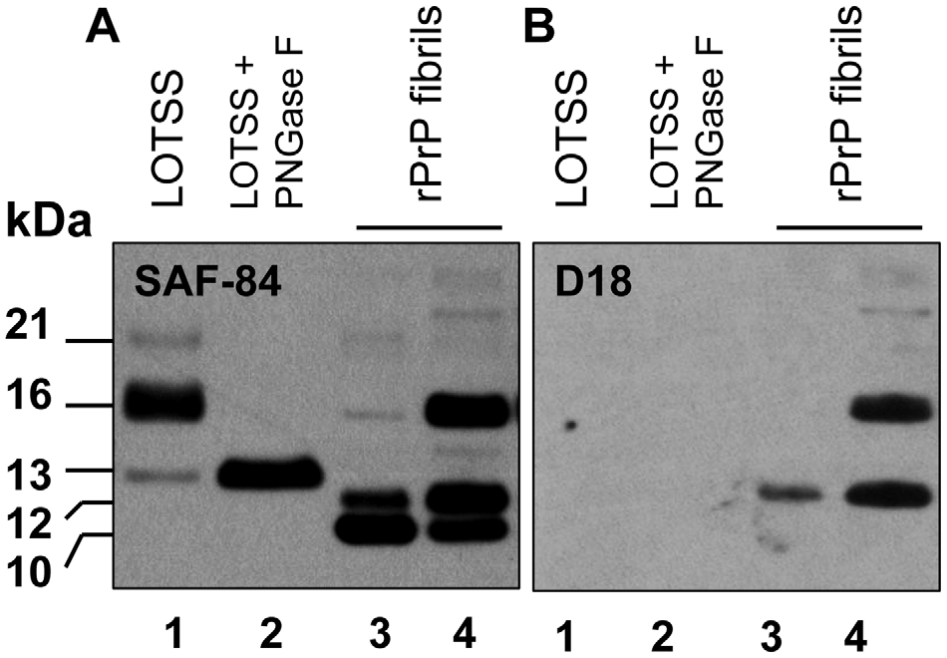
Analysis of the C-terminal PrPres fragments. Western blot of PK-digested BH from the animal inoculated with BSA-annealed rPrP amyloid fibrils (lanes 1, 2) or BSA-annealed rPrP amyloid fibrils (lanes 3 and 4) stained with SAF-84 (panel **A**) or D18 (panel **B**) antibodies. BH in lane 2 was treated with PNGase F. Before PK digestion, rPrP fibrils were mixed with 10% NBH to balance the condition for PK digestion. Samples in lanes 1, 2 and 3 were treated with 20 µg/ml of PK, and the sample in lane 4 – with 10 µg/ml of PK. Western blot was stained with SAF-84.

During serial transmission of LOTSS, we observed a dynamic transformation of the PK-resistant band pattern. PrPres consisted of atypical C-terminal fragments at the 1^st^ passage, and then atypical PrPres shifted to a predominantly 21–30 kDa PK-resistant core typical for PrP^Sc^ at the 3^rd^ passage (Figures 1B and 5). To determine whether the atypical PrPres encompasses the same region as the PK-resistant core of rPrP fibrils, BH from the animal of the 1^st^ passage was treated with PNGase F to remove carbohydrates. The PK-resistant core of annealed rPrP fibrils was previously found to consists of two major fragments of ∼138–231 and 152/153–231 [18], [25]. Upon treatment with PNGase F, the three PK-resistant bands of 21, 16 and 13 kDa merged into a single band of ∼13 kDa illustrating that these three bands represent di-, mono- and unglycosylated forms of a single PK-resistant fragment (Figure 8). This C-terminal fragment reacted with R1 (epitope 225–231) and SAF-84 (epitope 160–170), but not with 3F4 (epitope 109–112) or D18 (epitope 133–157) antibodies (Figures 1A, and 8A and B). Therefore, the atypical PrPres fragments matched the lower PK-resistant band of the fibrillar core that encompassed resides 152/153–231. The difference between PAGE-mobility of the rPrP 152/153–231 fragment and 13 kDa atypical PrPres fragment was likely due to a GPI-anchor in the last one. This result supports the hypothesis that the structure of LOTSS PrPres originated from rPrP fibrils.

Together with previous data (Figure 1), the results on epitope mapping illustrate that a dynamic transformation of LOTSS PrPres occurred during three serial passages. This transformation involved an increase in the size of the PK-resistant core from the atypical PrPres to the one typical for authentic PrP^Sc^. Notably, the change in size was accompanied by a significant shift in the ratio of three PrP glycoforms recruited by PrPres. While atypical PrPres preferred monoglycosylated PrP (16 kDa band, Figure 1B), standard PrPres at the 3^rd^ passage favored a diglycosylated PrP (Figure 1B and D), a feature common for all previously known hamster strains.

## Discussion

**Figure 9:**
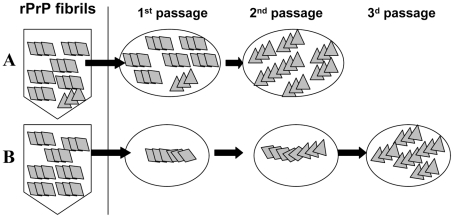
Schematic representation of two mechanisms responsible for generating transmissible prion diseases *de novo*. According to the first mechanism (**A**), the preparations of rPrP amyloid fibrils contain very small amounts of PrP^Sc^. The silent stage of the disease is attributed to the long time required for amplification of this extremely small amount of PrP^Sc^. A second mechanism referred to as deformed templating postulates that there are no PrP^Sc^ particles in the preparations of amyloid fibrils (**B**). Instead, when inoculated into animals, amyloid fibrils can seed conversion of PrP^C^ into PrPres, although with a low efficiency. PrPres undergoes slow transformation, a process that might require long silent stage, before authentic PrP^Sc^ emerges.

In the current work, transmissible prion disease was generated in wild type animals upon inoculation of rPrP amyloid fibrils produced *in vitro* in the absence of a cellular environment or co-factors. When triggered by recombinant fibrils, only a small fraction of animals showed signs of infection. Furthermore, it took two additional serial passages for a fully developed disease with a distinct set of symptoms to evolve. Two alternative models could be put forward to explain the low success rate in infecting the animals by rPrP fibrils (Figure 9). According to the first model, the preparations of rPrP amyloid fibrils contain very small amounts of PrP^Sc^ or particles with a structure of authentic PrP^Sc^ (Figure 9A). The second model proposes that the formation of PrP^Sc^ and emergence of transmissible prion diseases could be triggered in wild type animals by seeding material that lacks PrP^Sc^ (Figure 9B). This model takes into consideration previous data that showed that the structures of PrP^Sc^ and rPrP fibrils are fundamentally different as evident from X-ray diffraction analysis [14], [17], FTIR spectroscopy [26], [27], AFM and EM imaging and seeding-specificity assay [16]. Because of the structural differences, rPrP fibril-induced seeding of PrP^Sc^ is not efficient, which could explain the low rate of infection in the first passage. For the same reasons, transformation of the rPrP amyloid structure into PrP^Sc^ structure might involve several steps before fully infectious authentic PrP^Sc^ emerges, yielding the long silent stage to clinical disease.

The results of the current and previously published studies strongly support the second model. First, no PrP^Sc^ was found in the preparations of rPrP amyloid in an assay format that detects miniscule amounts of prions. One could argue that the efficiency of prion amplification and the sensitivity of detection in sPMCAb are strain-specific. We found that the efficiency of LOTSS PrP^Sc^ amplification in sPMCAb was similar to that of 263 K PrP^Sc^ (Figure 3SA). 100% and 83% of sPMCAb reactions seeded with asymptomatic LOTSS brains serially diluted 10^9^ or 10^10^-fold, respectively, were positive, whereas all reactions seeded with 10^11^-fold and higher dilutions were negative (Figure 7). This result suggests that the limiting dilution was reached in the experiment on sPMCAb titration. It also illustrates that 10^10^-fold diluted LOTSS brain material has only one or very few PMCAb-active PrP^Sc^ particles per 100 µl of the reaction volume, and that these quantities of LOTSS PrP^Sc^ could be effectively detected in our assay format. If one assumes that the first model is correct, the amount of infectivity per 50 µl of inoculum should be equivalent to approximately 0.5–1 infectious dose to account for the low infection rate in the first passage (3 out of 7 animals). This amount of infectivity is equivalent to ∼10,000–100,000 PrP molecules or to ∼100–1000 PrP^Sc^ particles, assuming that an average PrP^Sc^ particle consists of ∼100 PrP molecules [24]. This amount of PrP^Sc^ is well above the detection limits of sPMCAb, and should have been easily detected if present in preparations of rPrP fibrils.

Second, the experimental protocol used for producing rPrP amyloid fibrils employs denaturants (a mixture of 1M GdnHCl and 3M urea) - solvent conditions under which PrP^Sc^ undergoes denaturation. Because rPrP fibrils are much more conformationally stable than PrP^Sc^ [6], [21], [28], rPrP fibrils can be formed under solvent conditions where PrP^Sc^ is largely denatured. Furthermore, conversion of PrP^C^ into authentic PrP^Sc^ *in vitro* requires RNA and lipids [9], [10], whereas rPrP amyloid fibrils were formed in the absence of any cellular co-factors. Therefore, it is highly unlikely that PrP^Sc^ particles with authentic structures could be formed during the preparation of rPrP fibrils conducted in the absence of co-factors essential for authentic PrP^Sc^ structures and under solvent conditions that promote PrP^Sc^ denaturation.

Third, when transmissible prion disease is triggered by rPrP amyloid fibrils, a decrease in PrP^Sc^ conformational stability was observed during serial passages of synthetic prions [6], [29]. Similar dynamics in PrP^Sc^ conformational stability was observed regardless of whether transgenic mice or Syrian hamsters were used for inoculating rPrP fibrils suggesting that a common pathway in the genesis and evolution of transmissible prion protein structures might exist [6], [29]. Observed changes in the physical properties illustrate that PrP^Sc^ structure undergoes transformation during serial passages providing a direct support for the second model.

Fourth, the first model has difficulties in explaining two silent passages. While the first silent passage could be attributed to the low titer of infectious material in the preparation of rPrP fibrils, it is challenging to explain the second silent passage without assuming that the neurotoxic properties of LOTSS PrP^Sc^ are evolving slowly. Together with a dynamic change in PK-resistant profile, the second silent passage is indicative of transformation in PrPres structure and its neurotoxic features, which is consistent with the second model.

Fifth, significant quantitative and qualitative changes in PrP deposition were observed during LOTSS serial transmission. In animals from the 2^nd^ passage, LOTSS-specific PrP was predominantly found in the form of subpial and subependymal plaques, whereas animals from the 3^rd^ passage showed a dramatic increase in diffuse synaptic immunoreactivity and perineuronal deposition in addition to plaques. Furthermore, involvement of new brain regions positive for PrP immunostaining was observed during the serial passage of LOTSS, pointing out possible changes in strain neurotropism, which again is consistent with a dynamic transformation in PrPres biological and physical features. Previously, a change in the pattern of PrP accumulation was found to accompany strain adaptation during serial transmission in a new host [30]. The changes in neurotropism or distribution pattern could not be attributed solely to an increase in the total amounts of LOTSS PrPres, because certain brain regions including frontal deeper layers, hippocampus and granular cerebellum displayed a disproportionally high increase in the amounts of LOTSS-specific PrP deposition in the 3^rd^ passage despite a shorter life span.

Sixth, in recent studies on synthetic prions, a strong correlation between conformational stability of rPrP amyloid fibrils, the stability of PrP^Sc^ produced in animals upon inoculating rPrP fibrils and the incubation time to disease was described [5]. If a miniscule fraction in the preparation of rPrP fibrils is responsible for the disease, the correlation between stability of rPrP amyloid, which is a bulk property of fibril preparation, and PrP^Sc^ would be challenging to explain. Again, these results provide a strong support for the second model.

Seventh, as judged from clinical and neuropathological features, both synthetic strains generated *de novo* upon inoculating Syrian hamsters with rPrP fibrils (LOTSS and SSLOW) were remarkably different from all previously known rodent strains including those generated in sPMCA [8]–[10]. Among the most distinguishable LOTSS and SSLOW features were the long incubation time to and slow progression of clinical disease, accumulation of large plaques in subpial and subependymal areas, distinctive lesion and PrP immunoreactivity profiles and unusual clinical phenotype (obesity, hair loss). The fact that SSLOW and LOTSS can be separated into a distinct class of prion strains remarkably different from strains produced in sPMCA or isolated from animals is consistent with the hypothesis that SSLOW or LOTSS PrP^Sc^ originated from unique structures or rPrP fibrils.

Eighth, observation of the atypical, C-terminal, low molecular weight PK-resistant products in the first passage strongly supports the last conclusion. Both, atypical PrPres and rPrP fibrils had very similar if not identical PK-resistant cores that encompassed epitopes for SAF-84 and R1, but not 3F4 antibodies [18], [25]. This result supports the idea that the structure of fibrillar rPrP gave origin to LOTSS PrPres.

Ninth, the transformation in LOTSS PrPres structure is demonstrated by an increase in the size of the PK-resistant core from atypical to standard and the change in glycosylation preferences. This transformation provides direct support for the second model. While atypical PrPres predominantly recruited monoglycylated PrP, the LOTSS PrP^Sc^ preferred diglycosylated PrP, a feature which is common for all currently known hamster-adapted prion strains.

All the data on synthetic prions accumulated to date support the hypothesis that transmissible prion diseases can be triggered by PrP structures substantially different from that of authentic PrP^Sc^ and in the absence of PrP^Sc^ in preparation of rPrP fibrils (Figure 9 B). This hypothesis assumes that only a partial overlap or distant similarities in structures of fibrillar rPrP and PrP^Sc^ are sufficient for triggering transmissible prion diseases in animals. While the precise mechanistic details for inducing PrP^Sc^ formation by conformationally different PrP structures remain to be elucidated, the model of conformational switching provides one possible explanation of how such transformation occurs [31], [32]. According to this model, the global folding pattern of PrP molecules within amyloid fibrils and PrP^Sc^ are different, nevertheless, they share a common structural motif. For instance, a common β-strand that can link two structures provides opportunity for limited templating. This model is consistent with experimental observations that the global structures of PrP^Sc^ and rPrP fibrils are different [14], [15], [17], and explains that there is a correlation between conformational stability of two structures [5]. Because of only partial overlap between the two structures, the seeding of PrP^Sc^ by rPrP fibrils is inefficient, which explains the low infection rate in the first passage. Previous studies on molecular imaging of single amyloid fibrils provided a proof of principle that the conformational switching between two alternative PrP folding patterns can occur within an individual PrP fibril or particle [31].

The current study proposes that a new mechanism designated as ‘deformed templating’ that is different from the classical templating might exist. In classical templating, the folding pattern of a newly recruited polypeptide chain accurately reproduces that of the template. In deformed templating, while the template provides limited seeding, a newly recruited polypeptide chain acquires a new folding pattern which only partially overlaps with the folding pattern of the template. Two glycosyl groups and a GPI anchor present in PrP^C^ might impose steric constraints on the spectrum of folding patterns available to PrP^C^, therefore providing a driving force behind switching PrP folding patterns from rPrP fibril-specific to PrP^S^-specific. In fact, the evolution of PrP^Sc^ structure might involve at least two steps. The first step involves a transformation of the fibrillar rPrP structure to the structure of atypical PrPres, which is characterized by a short PK-resistant core, a preference for monoglycosylated PrP, and inactivity in PMCAb. We do not know how long it took for the transition from fibrillar rPrP to atypical PrPres to occur in animals. The second step is a transformation from atypical PrPres to LOTSS PrP^Sc^ that displays a standard PK-resistant core, favors diglycosylated PrP, is PMCAb-active and shows RNA-dependency in sPMCAb amplification.

If the hypothesis that a fibril-specific PrP folding pattern can template a distinct folding pattern within PrP^Sc^ is correct, one can assume that the opposite reaction, i.e. the seeding of rPrP fibrils by PrP^Sc^ is also possible. Indeed, several assays exploited the phenomenon of PrP^Sc^-seeded conversion of α-rPrP into amyloid fibrils for detecting miniscule amounts of PrP^Sc^ [33], [34]. Interestingly, the structure of rPrP fibrils produced as a result of seeding by PrP^Sc^ only distantly resembled those of PrP^Sc^ structure and had limited infectivity [35].

Although a number of unique features warrant classification of SSLOW and LOTSS into a separate class of prion strains, PrP^Sc^ of both synthetic strains was predominantly diglycosylated, and PMCAb amplification of both strains showed strong RNA dependency, features that were common with other hamster strains [22], [23]. Interestingly, the RNA dependency in LOTSS amplification emerged during the first passage (Figure 1C), whereas the first clinical signs were observed only during the 3^d^ passage. These results suggest that the two stages in the evolution of PrP^Sc^ structure – (1) transformation of fibrillar structure, which is RNA independent, into a structure that relies on RNA in self-replication and (ii) acquiring neurotoxic features essential for clinical disease, are separated. Dramatic increase in diffuse synaptic PrP immunoreactivity at the 3^rd^ passage suggests that the clinical expression of the disease might require synaptic PrP deposition. The mechanism underlying significant changes in the PrP deposition pattern during LOTSS serial transmission and possible connections between changes in PrP^Sc^ structure and its deposition pattern remain to be explored in future studies.

Inter-species prion transmission to new hosts including human is known to involve a long clinically silent stage that accompanies an adaptation to a new host [36], [37]. In the case of inter-species transmission, the long silent stage, which is referred to as a transmission barrier, is attributed at least in part to the differences in amino acid sequence of host PrP^C^ and donor PrP^Sc^. Moreover, the cross-species barrier could also be due to change in preferences for species-specific cofactors in prion replication [23]. Despite identity in the amino acid sequence of the rPrP inoculum and host PrP^C^, the silent stage in the current studies was found to be very long and consisted of two passages. Such a long silent stage is presumably attributable to the evolution of PrP^Sc^ physical and biological features and appears to be more prominent than those associated with inter-species transmission despite the fact that no change in the same amino acid sequence was involved. Consistent with this view, FTIR and X-ray diffraction analysis revealed that PrP^Sc^ isolated from different species display very similar folding patterns, which appear to be fundamentally different from the PrP folding pattern within rPrP fibrils [17], [26], [27], [38].

In other studies on inter-species transmission including the work on transmission of BSE to wild type mice or transmission of the hamster-adapted strain 263 K to transgenic mice carrying murine *PRNP* gene with a mutation P101L [39], [40], the clinical disease was observed in the first passage but in the absence of detectable amounts of PrPres. In the current studies, the second passage of LOTSS produced large amounts of PrPres, however, without any detectible clinical symptoms. Both sets of data were consistent with the emerging hypothesis that infectious and neurotoxic PrP species might be two different entities [41].

Much of the public health risk from prion diseases derives from their long asymptomatic incubation times [42]. During the asymptomatic period humans pose a real risk of spreading the infection through invasive medical procedures or tissue or blood donation [43]. The current work raises the importance of using antibodies against several PrP regions including the C-terminal region for effective diagnosis of prion diseases. Noteworthy, C-terminal PK-resistant fragments with a length similar to that of atypical PrPres described here were found in a majority of individuals with sporadic CJD [44]. If asymptomatic stages are accompanied by accumulation of predominantly atypical PrPres, while the procedures that rely only on N-terminal antibodies such as 3F4 are employed, the pathogenic process would remain undetected. This study raises a great concern that transmissible prion diseases can be triggered by protein structures which are considered to be not-infectious. Furthermore, spread of transmissible, disease-related prion structures can occur undetected during a silent stage of prion evolution when no clinical expression of the disease can be observed.

The hypothesis that transmissible prion diseases can be triggered by cross-β PrP structures substantially different from that of authentic PrP^Sc^ has large implications for understanding the ethiology of prion and, possibly, other neurodegenerative diseases. Several recent studies documented that amyloid forms of several proteins linked to neurodegenerative diseases were capable of seeding their own aggregation in a prion-like manner in a cell and spreading from cell to cell through the nervous system (reviewed in [45]–[47]). The recent study provided the first illustration that pathological changes associated with non-prion neurodegenerative diseases could be induced or transmitted through inoculation of the aggregated forms of non-prion proteins such as Aβ [48]. Furthermore, a growing amount of evidence illustrates that amyloids can template structures different from their own [31]. It is generally assumed that self-perpetuating aggregation requires identity in amino acid sequence between seeds and substrate. An increasing number of studies, however, suggest the possibility of cross-talk between non-related amyloidogenic proteins [49]–[51]. *In vivo,* amyloidosis of one protein was found to be triggered by fibrils of an unrelated protein in a manner similar to cross-seeded polymerization [49]–[51]. Cross-talk between several yeast prion proteins provides another example of how direct interactions between newly forming and pre-existing heterologous fibrils might take place in a cell [52]–[54]. Moreover, pathological studies revealed that protein aggregates produced from two different proteins or peptides, including PrP, Aβ, α-synuclein, immunoglobulin light chain λ, and β_2_ microglobulin often co-localize within the same amyloid plaques in a variety of organs or tissues [55]–[59]. The promiscuous nature of the propagating activity of amyloid structures can lead to devastating consequences. For instance, cross-talk between non-related amyloidogenic proteins may offer a possible explanation for development of the age-related conformational disorders that are considered to be sporadic. It would be interesting in future studies to define the spectrum of structures and sequences capable of triggering PrP^C^ to PrP^Sc^ conversion and induce transmissible prion diseases.

## Materials and Methods

### Ethics statement

This study was carried out in strict accordance with the recommendations in the Guide for the Care and Use of Laboratory Animals of the National Institutes of Health. The protocol was approved by the Institutional Animal Care and Use Committee of the University of Maryland, Baltimore (Assurance Number A32000–01; Permit Number: 0309001).

### Expression and purification of rPrP and formation of rPrP fibrils

Syrian hamster full-length recombinant PrP encompassing residues 23–231 (rPrP) was expressed and purified according to the previously described procedure [60] with minor modifications [6]. Lyophilized rPrP was dissolved in 6 M GdnHCl to prepare a 3 mg/ml stock solution of rPrP. To form fibrils, the rPrP stock solution was diluted to a final protein concentration of 0.5 mg/ml and incubated at 37°C in 20 mM sodium acetate (pH 5.0), 1 M GdnHCl, 3 M Urea, 150 mM NaCl, 10 mM EDTA for 4 days with continuous horizontal shaking at 600 rpm. Amyloid formation was confirmed by increased Thioflavin T fluorescence and electron microscopy [60]. Fibrils were dialyzed into 10 mM sodium acetate, pH 5.0.

To perform annealing, rPrP fibrils at 0.1 mg/ml were heat-treated in PBS, pH 7.4, in the presence of 5 mg/ml BSA (Sigma, catalog #A3294) and 0.1% Triton X-100 using the following procedure: 50 µl aliquots in thin-wall PCR tubes with domed caps were placed into the PCR machine and subjected to 5 cycles of 1 min incubation at 80°C followed by 1 min incubation at 37°C. All aliquots were pooled together and the combined volume was immediately transferred to the animal facility for inoculation.

### Bioassay

For the first passage, weanling Syrian hamsters were inoculated intracerebrally with the preparation of rPrP fibrils described above. Each animal was anesthetized with interperitoneal pentobarbital before receiving 50 µl of inoculum. Hamsters were observed daily for disease starting from the third month postinoculation. They were euthanized at 661 days post inoculation without any signs of clinical disease, their brains removed aseptically and saved for subsequent passage and analysis.

For the second and third passages, 10% BHs prepared by sonication in PBS, pH 7.4 (see below), were dispersed by an additional 30 sec of sonication immediately before inoculation. Each hamster received 50 µl of inoculum intracerebrally under general anesthesia (2% O_2_/4 MAC isoflurane). After inoculation, hamsters were observed daily for disease.

### Proteinase K digestion

Brains were collected aseptically and cut in half with disposable scalpels. One half was used to prepare 10% BHs, while the second half was stored at −80°C for future analysis or fixed in formalin for histopathology. Homogenization was performed on ice in PBS, pH 7.4, with a Sonics vibra cell VCX750 (Newtown, CT) tip sonicatior fitted with a stepped microtip by 2 pulses of 30 sec sonication with 30 sec cooling on ice between the pulses. For the Proteinase K digestion in sarcosyl, an aliquot of 10% brain homogenate was mixed with an equal volume of 4% sarcosyl in PBS, supplemented with 50 mM Tris, pH 7.5, and digested with 20 µg/ml PK for 30 min at 37°C with 1000 rpm shaking (Eppendorf Thermomixer). Alternatively, 1% brain homogenate in conversion buffer was supplemented with 0.25% SDS and 50 µg/ml PK and digested at 37°C for 1 hour. Proteinase K digestion was stopped by adding SDS sample buffer and heating the samples for 10 min in a boiling water bath. After loading onto NuPAGE 12% BisTris gels and transfer to PVDF membrane, PrP was detected with 3F4 (epitope 109–112), D18 (epitope 133–157), SAF-84 (epitope 160–170), or R1 (epitope 225–231) antibody, as indicated. For PK digestion of recombinant fibrils annealed in BSA, fibrils were diluted to 10 µg/ml in 1% NBH supplemented with 0.25% SDS and digested with 10 or 20 µg/ml PK for 1 hour at 37°C.

### Deglycosylation of PrPres

Removal of N-linked glycans was performed as previously described [61], using glycerol free PNGase F and supplied buffers (New England BioLabs). 5% BH in sarcosyl was digested with 20 µg/ml Proteinase K as described above. After termination of PK digestion with 2 mM PMSF, the resulting 36 µl of digest was supplemented with 4 µl of 10x glycoprotein buffer and heated at 95°C for 10 min. After heat-treatment, 5 µl of 10xG7 reaction buffer, 5 µl of 10% NP-40, and 3 µl of PNGase F enzyme were added, and the samples were incubated overnight at 37°C. The next day, the samples were boiled for 10 min in SDS sample buffer (Invitrogen) and loaded onto NuPAGE 12% BisTris gels. After transfer to PVDF, PrP was detected with SAF-84 or D18 antibody as indicated.

### Conformational stability assay

10% brain homogenates were sonicated for 30 sec at 50% power efficiency within Misonix-4000 microplate horn filled with 350 ml water. Then the samples were diluted to 1% BH with conversion buffer and incubated with various concentrations of GdnHCl within 96 Deep Well 1 ml plate (Fisher #12–566–120) for 1 hour at room temperature. After incubation, all samples were diluted with PBS to A final concentration of GdnHCl of 0.4 M and incubated for an additional 1 h at room temperature. The incubation was followed by loading 50 µl of each denaturation point onto a nitrocellulose membrane using a BioDot microfiltration apparatus (Bio-Rad) according to the manufacturer' manual. After two washes with PBS, the membrane was removed from the apparatus, immediately blocked with 2.5% non-fat milk in PBST for 30 min, incubated with antibody overnight at +4°C, and developed according to the common immunodetection protocol.

To compare relative exposure of 3F4 and SAF-84 epitopes in LOTSS, SSLOW and 263 K, equal amounts of brain homogenates were denatured with 5 M GdnHCl and loaded onto nitrocellulose membrane with a BioDot apparatus. To eliminate the possibility of PrP^C^ influencing the data, the relative exposure of epitopes was also analyzed after mild denaturation with 1 M GdnHCl and PK digestion. Chemiluminescent signals were measured by a Typhoon 9200 variable mode imager.

### Protein misfolding cyclic amplification

Healthy hamsters were euthanized and immediately perfused with PBS, pH 7.4, supplemented with 5 mM EDTA. Brains were dissected, 10% brain homogenate (w/v) was prepared using ice-cold conversion buffer and glass/Teflon tissue grinders cooled on ice and attached to a constant torque homogenizer (Heidolph RZR2020). The brains were ground at low speed until homogeneous, then 5 additional strokes completed the homogenization. The composition of conversion buffer was as previously described [62]: Ca^2+^-free and Mg^2+^-free PBS, pH 7.5, supplemented with 0.15 M NaCl, 1.0% Triton, and 1 tablet of Complete protease inhibitors cocktail (Roche, Cat# 1836145) per 50 ml of conversion buffer. The resulting 10% NBH in conversion buffer was precleared by 2 min centrifugation at 500 g and the resulting supernatant was used as the substrate in PMCA with beads (PMCAb) reactions [19]. 100 µl samples in 0.2 ml thin-wall PCR tubes containing 3 Teflon 3/32”beads (McMaster-Carr #9660 K12) were placed in a floating rack inside Misonix-4000 microplate horn sonicator, filled with 350 ml water. Two coils of rubber tubing attached to a circulating water bath were installed for maintaining 37°C inside the sonicator chamber. The standard sonication program consisted of 30 sec sonication pulses delivered at 50% power efficiency applied every 30 min during a 24 hours period, therefore, each round consisted of 48 cycles. For each subsequent round, 10 µl of the reaction from previous round were added to the 90 µl of a fresh substrate. To produce RNA-depleted BH, precleared NBH was incubated with 100 µg/ml RNase A (Sigma, Cat. # R-4875) for 1 hour at 37°C prior to its use as a substrate in PMCAb. Complete digestion was confirmed using RNA content analysis in 1.2% Agarose gel and ethidium bromide staining.

For the titration experiment, 100 µl aliquots of 10% BH were sonicated with 3 Teflon 3/32” beads for 30 sec in PMCA conditions before serial 10-fold dilutions into conversion buffer. sPMCAb titration was done for two animals from the 2^nd^ passage of LOTSS. To determine PMCAb_50_ values, nonlinear least-square regression analysis of the data to the following equation was performed in Sigma Plot:
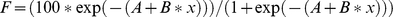
where *F* is percent of positive PMCAb reactions, *x* is the logarithm of the dilution fold, and *A* and *B* are two fitting parameters that define the position of a limiting dilution transition on the *x* axis and the slope of the transition, respectively. PMCAb_50_ was calculated according to the equation:
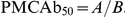



To analyze production of PK-resistant PrP material in PMCA, 10 µl of the sample were supplemented with 5 µl SDS and 5 µl PK, to a final concentration of SDS and PK of 0.25% and 50 µg/ml respectively, followed by incubation at 37°C for 1 hour. The digestion was terminated by addition of SDS-sample buffer and heating the samples for 10 min in boiling water bath. Samples were loaded onto NuPAGE 12% BisTris gels, transferred to PVDF membrane, and probed with 3F4 or SAF-84 antibodies.

### Histopathological studies

Formalin fixed brain halves divided at the midline (right hemisphere), spinal cord, and spleen, were processed for hematoxylin-eosin stain as well as for immunohistochemistry for PrP, using the mouse monoclonal anti-PrP antibody 3F4 (1∶1000, Covance, Berkeley, CA, USA) and anti-glial fibrillar acidic protein (GFAP; 1∶3000, Dako, Glostrup, Denmark). Blocks were treated in formic acid (96%) prior to embedding in paraffin. For detection of disease-associated PrP, we applied a pretreatment of 30 minutes hydrated autoclaving at 121°C followed by 5 minutes in 96% formic acid. We evaluated all tissues for the presence of inflammation and PrP immunoreactivity, and the brain for the presence of spongiform change and degree of gliosis. Degree of spongiform change, neuronal loss and gliosis, and intensity of PrP immunostaining were semiquantitatively evaluated (0: none; 1: mild; 2: moderate; 3 severe) in the following anatomical regions as previously described: frontal cortex, hippocampus, caudate-putamen, thalamus, brainstem and cerebellum [6]. Lesion profiles were obtained by averaging scores of spongiform change, neuronal loss, and gliosis for each anatomical region and animal group.

## Supporting Information

Figure S1 **Analysis of BH from animals inoculated with rPrP fibrils using sPMCAb.** 10% BH from the animals inoculated with BSA-annealed rPrP amyloid fibrils was diluted 10-fold into 10% NBH and subjected to two (panel **A**) or six (panel **B**) sPMCAb rounds. BH from animal #2 showed atypical PK-resistant bands (Figure 1), from animal #6 showed PrPres after one PMCAb round and from animal #7 - after six sPMCAb rounds. Ten reactions, each consisted of six sPMCAb rounds, were conducted in non-seeded NBHs as negative controls (panel **C**). Each PMCAb round consisted of 48 cycles, 30 min each; 10-fold dilutions were used for each subsequent sPMCAb round. Western blot was stained with 3F4.
(TIF)

Figure S2 **Analysis of BH from LOTSS-inoculated animals of the 2^nd^ passage.** Western blotting of the BHs from the animals of 2^nd^ passage of LOTSS stained with 3F4 (top panel) or SAF-84 (bottom panel). Animals # 1–5 and 7 showed large amounts of standard and atypical PrPres (lanes 1–5 and 7, respectively), whereas the animal #6 predominantly shows atypical PrPres. BH from animal # 6 (lane 6) was subjected to a single PMCAb round (lane 9).
(TIF)

Figure S3 **Analysis of LOTSS PrP^Sc^ amplification rate in PMCAb and resistance to PK. (A)** BH from LOTSS- (top panel) or 263 K-inoculated animals (lower panel) were diluted 10^4^-fold into 10% NBH and subjected to four sPMCAb rounds. The material amplified in each round was diluted 30-, 100-, 300-, or 1000-fold into 10% NBH for the next PMCAb round, as indicated. Undigested 10% NBH is provided as a reference. (**B**) BHs from LOTSS- (top panel) or 263 K-inoculated animals (bottom panel) were diluted to 1% in conversion buffer and treated with increasing concentration of glycerol-free Proteinase K (Sigma #P6556) in the presence of 0.25% SDS for 1 hour at 37°C. Western blots were stained with 3F4. BH from the 2^nd^ passage of LOTSS was used in both experiments.
(TIF)

Figure S4 **Analysis of conformational stability.** 1% BH from animals inoculated with 263 K (panel **A**), SSLOW (panel **B**), or LOTSS (panel **C**) was incubated with increasing concentrations of GdnHCl from 0.4 to 4 M for 1 h, as indicated, then diluted out of GdnHCl, equilibrated for 1 h at room temperature and digested with 20 µg/ml PK, followed by addition of 2 mM PMSF and precipitation with 4 volumes of cold acetone. Undigested brain material exposed to 0.4 M GdnHCl is provided as a reference. BHs from the 2^nd^ passages of SSLOW or LOTSS were used. Western blotting was stained with 3F4.
(TIF)

Figure S5 **Analysis of strain-specific SAF-84 to 3F4 immunoreactivity ratio.** To compare relative immunoreactivity of SAF-84 and 3F4 epitopes, BHs from LOTSS-, SSLOW- or 263 K-inoculated animals were denatured with 5 M GdnHCl and loaded onto nitrocellulose membrane with BioDot apparatus (see methods) and stained with SAF-84 or 3F4 (panel **A**). To eliminate the possibility of PrP^C^ influencing the results, we also compared relative exposure of SAF-84 and 3F4 epitopes after mild denaturation of BHs with 1 M GdnHCl followed by PK digestion (panel **B**). Both methods show that the intensity ratio of SAF-84 to 3F4 in LOTSS-infected BH was significantly higher than that in SSLOW- or 263 K-infectd BH. BHs from the 2^nd^ passages of SSLOW or LOTSS were used. Data represent average ± SD of three replicas.
(TIF)

Table S1 **Bioassay of rPrP amyloid fibrils in golden Syrian hamsters and control experiments.**
(DOC)
