# Supplementary figures and images for: Genesis of Mammalian Prions: From Non-infectious Amyloid Fibrils to a Transmissible Prion Disease
Source: PLoS Pathog. 2011 Dec 1;7(12):e1002419. doi: 10.1371/journal.ppat.1002419 (PMC3228811; doi:10.1371/journal.ppat.1002419)

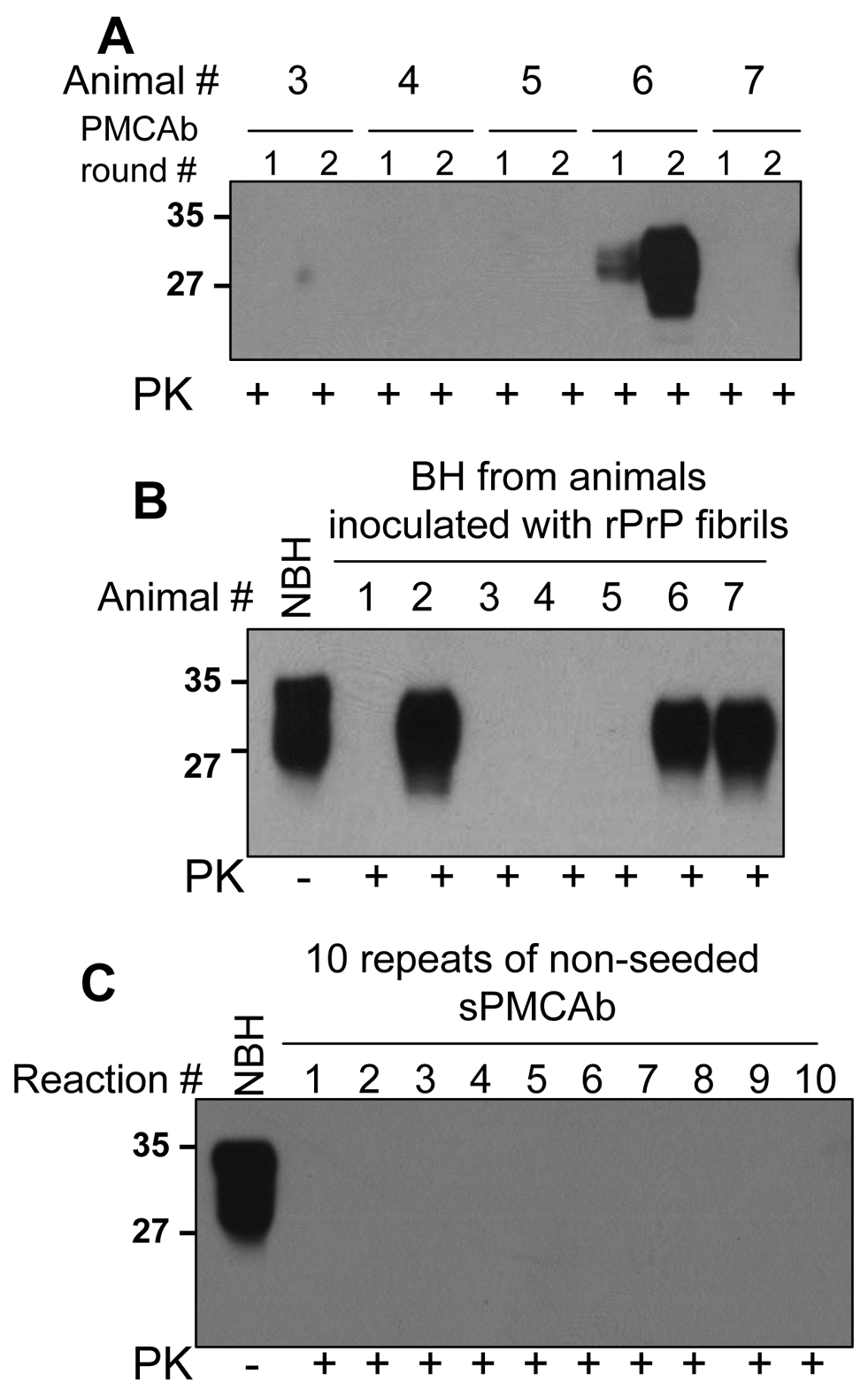

Supplement: Figure S1 — Analysis of BH from animals inoculated with rPrP fibrils using sPMCAb. 10% BH from the animals inoculated with BSA-annealed rPrP amyloid fibrils was diluted 10-fold into 10% NBH and subjected to two (panel A) or six (panel B) sPMCAb rounds. BH from animal #2 showed atypical PK-resistant bands (Figure 1), from animal #6 showed PrPres after one PMCAb round and from animal #7 - after six sPMCAb rounds. Ten reactions, each consisted of six sPMCAb rounds, were conducted in non-seeded NBHs as negative controls (panel C). Each PMCAb round consisted of 48 cycles, 30 min each; 10-fold dilutions were used for each subsequent sPMCAb round. Western blot was stained with 3F4. (TIF) [file ppat.1002419.s001.tif]

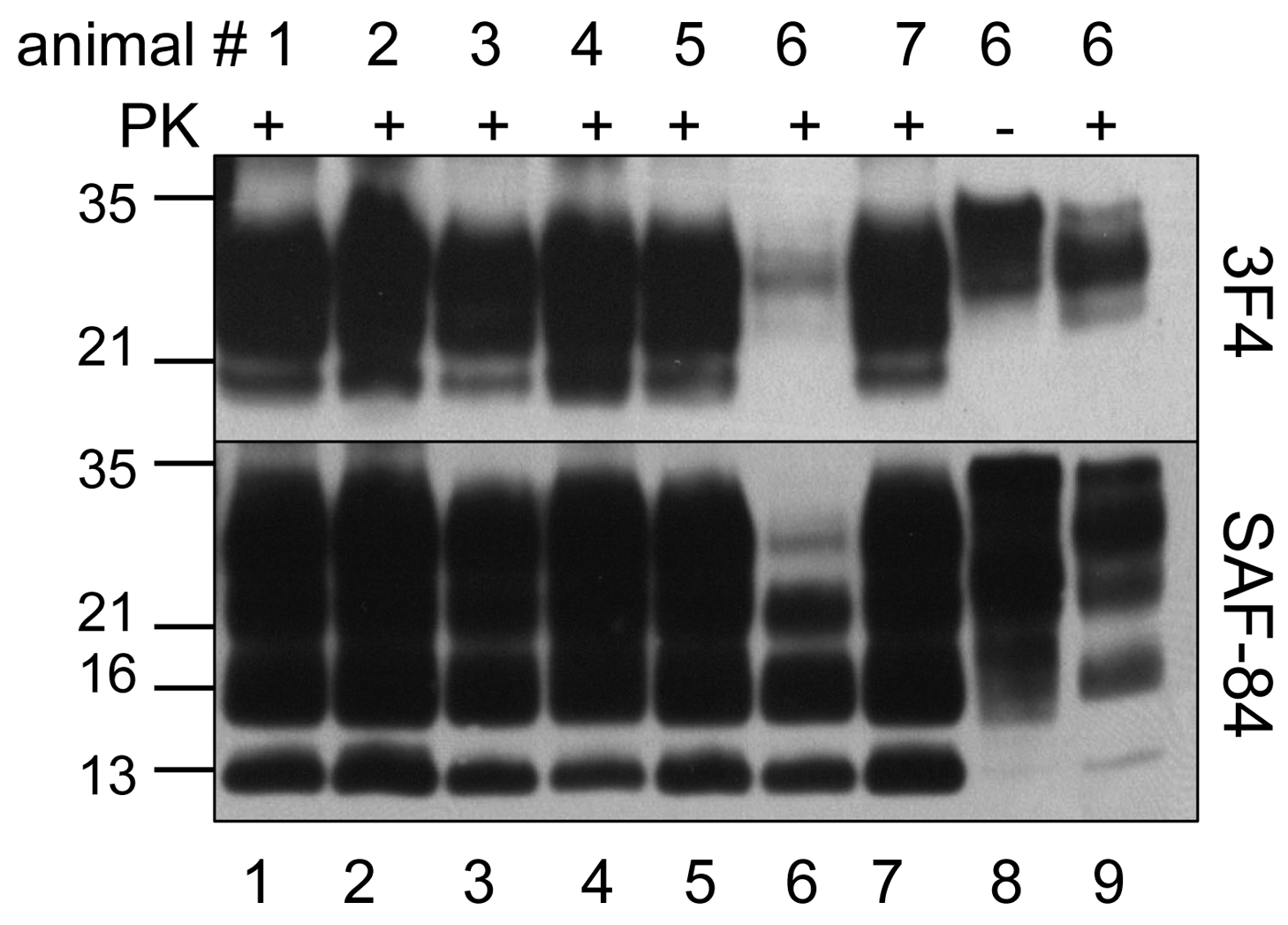

Supplement: Figure S2 — Analysis of BH from LOTSS-inoculated animals of the 2nd passage. Western blotting of the BHs from the animals of 2nd passage of LOTSS stained with 3F4 (top panel) or SAF-84 (bottom panel). Animals # 1–5 and 7 showed large amounts of standard and atypical PrPres (lanes 1–5 and 7, respectively), whereas the animal #6 predominantly shows atypical PrPres. BH from animal # 6 (lane 6) was subjected to a single PMCAb round (lane 9). (TIF) [file ppat.1002419.s002.tif]

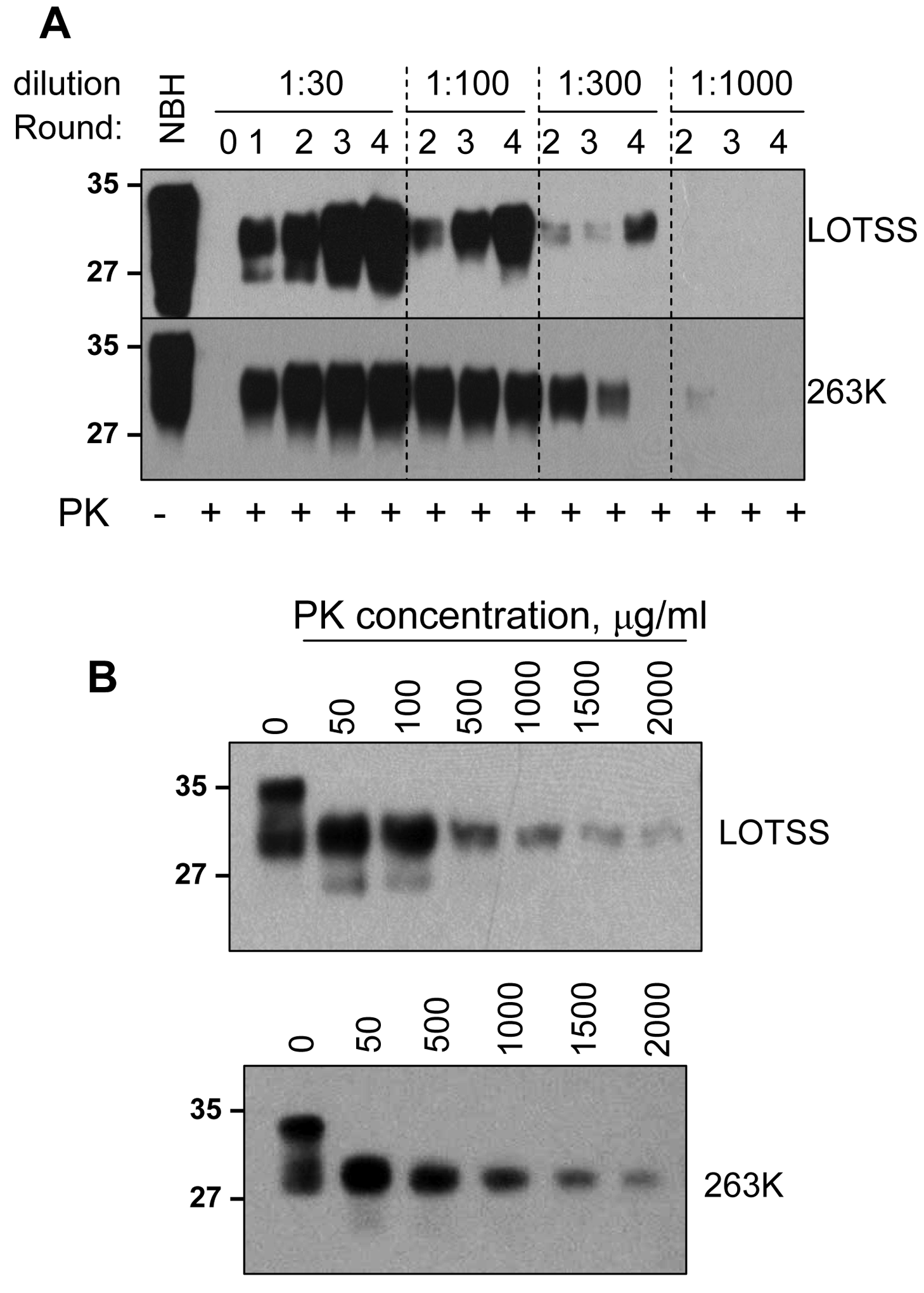

Supplement: Figure S3 — Analysis of LOTSS PrPSc amplification rate in PMCAb and resistance to PK. (A) BH from LOTSS- (top panel) or 263 K-inoculated animals (lower panel) were diluted 104-fold into 10% NBH and subjected to four sPMCAb rounds. The material amplified in each round was diluted 30-, 100-, 300-, or 1000-fold into 10% NBH for the next PMCAb round, as indicated. Undigested 10% NBH is provided as a reference. (B) BHs from LOTSS- (top panel) or 263 K-inoculated animals (bottom panel) were diluted to 1% in conversion buffer and treated with increasing concentration of glycerol-free Proteinase K (Sigma #P6556) in the presence of 0.25% SDS for 1 hour at 37°C. Western blots were stained with 3F4. BH from the 2nd passage of LOTSS was used in both experiments. (TIF) [file ppat.1002419.s003.tif]

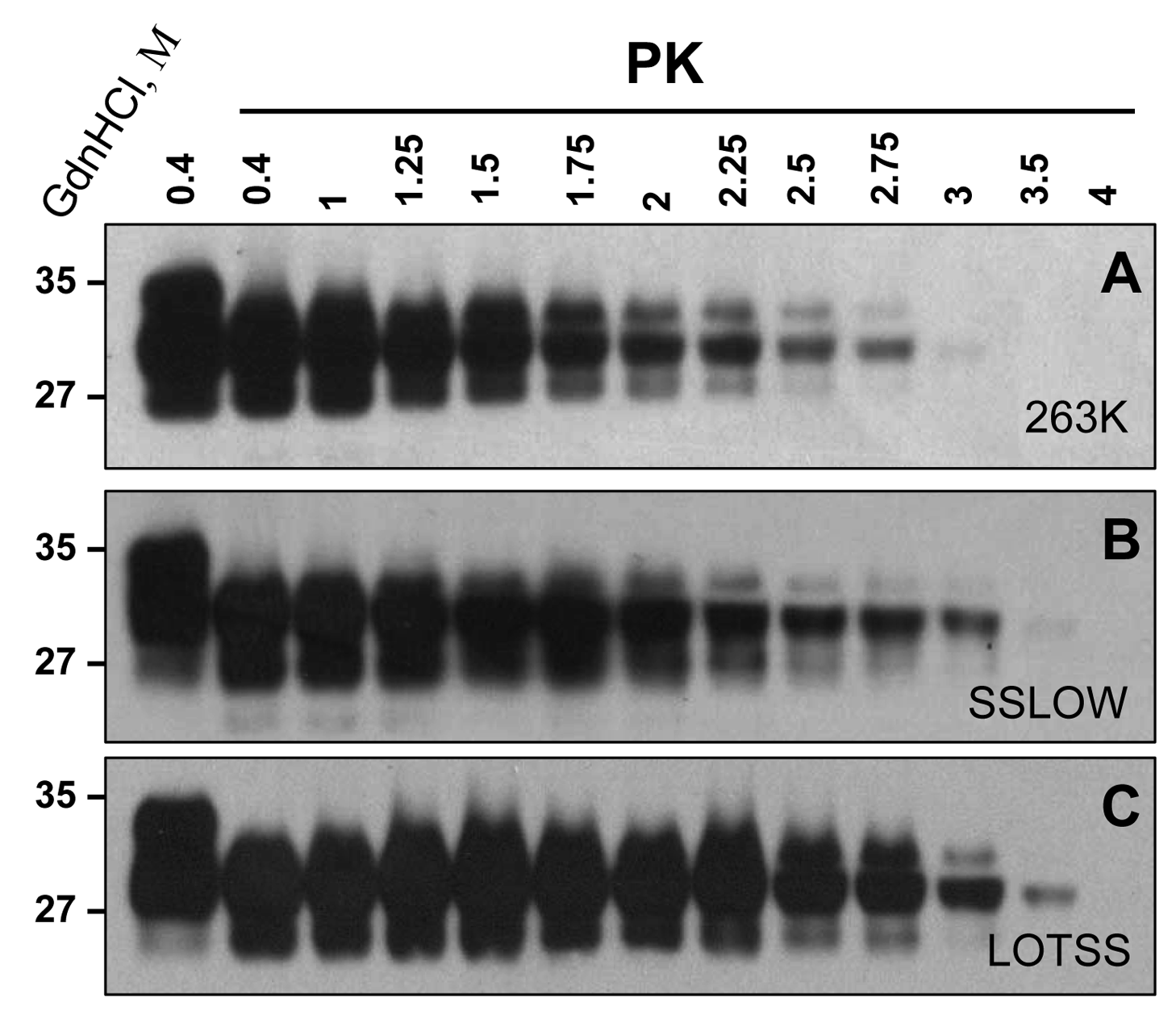

Supplement: Figure S4 — Analysis of conformational stability. 1% BH from animals inoculated with 263 K (panel A), SSLOW (panel B), or LOTSS (panel C) was incubated with increasing concentrations of GdnHCl from 0.4 to 4 M for 1 h, as indicated, then diluted out of GdnHCl, equilibrated for 1 h at room temperature and digested with 20 µg/ml PK, followed by addition of 2 mM PMSF and precipitation with 4 volumes of cold acetone. Undigested brain material exposed to 0.4 M GdnHCl is provided as a reference. BHs from the 2nd passages of SSLOW or LOTSS were used. Western blotting was stained with 3F4. (TIF) [file ppat.1002419.s004.tif]

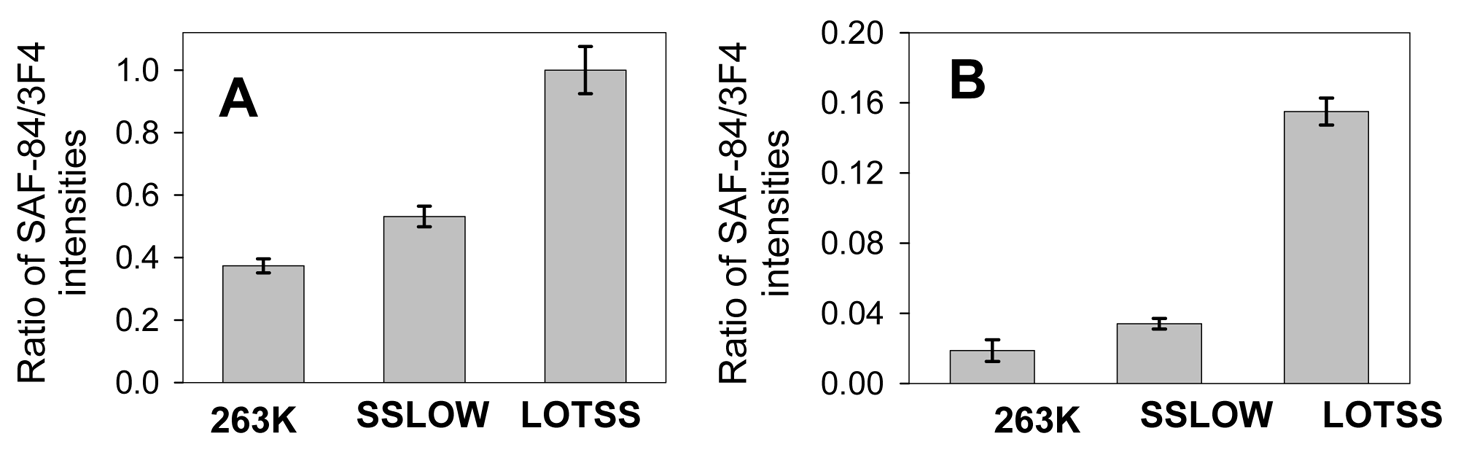

Supplement: Figure S5 — Analysis of strain-specific SAF-84 to 3F4 immunoreactivity ratio. To compare relative immunoreactivity of SAF-84 and 3F4 epitopes, BHs from LOTSS-, SSLOW- or 263 K-inoculated animals were denatured with 5 M GdnHCl and loaded onto nitrocellulose membrane with BioDot apparatus (see methods) and stained with SAF-84 or 3F4 (panel A). To eliminate the possibility of PrPC influencing the results, we also compared relative exposure of SAF-84 and 3F4 epitopes after mild denaturation of BHs with 1 M GdnHCl followed by PK digestion (panel B). Both methods show that the intensity ratio of SAF-84 to 3F4 in LOTSS-infected BH was significantly higher than that in SSLOW- or 263 K-infectd BH. BHs from the 2nd passages of SSLOW or LOTSS were used. Data represent average ± SD of three replicas. (TIF) [file ppat.1002419.s005.tif]
