# Supplementary material for: Genesis of Mammalian Prions: From Non-infectious Amyloid Fibrils to a Transmissible Prion Disease
Source: PLoS Pathog. 2011 Dec 1;7(12):e1002419. doi: 10.1371/journal.ppat.1002419 (PMC3228811; doi:10.1371/journal.ppat.1002419)
Supplement: Table S1 — Bioassay of rPrP amyloid fibrils in golden Syrian hamsters and control experiments. (DOC) [file ppat.1002419.s006.doc]

Table S1. Bioassay of rPrP amyloid fibrils in golden Syrian hamsters and control experiments

| Inoculum | n_s_/n_t_* | n_PK-res_/n_t_** | Euthanized at days PI |
| --- | --- | --- | --- |
| BSA-annealed rPrP fibrils | 0/7 | 3^(1)^ / 7 | 661 |
| None (age-matched controls) | 0/9 | 0/9 | 661^(3)^ |
| α-rPrP-monomer mock-annealed with NBH | 0/8 | 0/8 | 660 |
| 2^nd^ serial passage of LOTSS: 10% BH containing LMW PrPres | 0/7 | 7/7 | 661 |
| 2^nd^ serial passage: 10% BH from age-matched control #1 | 0/5 | 0/5 | 661 |
| 2^nd^ serial passage: 10% BH from age-matched control #2 | 0/5 | 0/5 | 661 |
| 3^d^ passage of LOTSS (animal #1) | 5/5 | 5/5 | 3 at 485; 494; 521 |
| 3^d^ serial passage of LOTSS (animal #2) | 7/7 | 7/7 | 471; 2 at 494; 521; 521; 546; 568 |
| 3^d^ serial passage: 10% BH from age-matched control #1 | 0/8 | 0/8 | 664 |

* - number of animals with clinical signs over the total number of animals survived to the end of the experiment.

** - number of animals with PrPres in BHs by Western blot or after PMCAb over the total number of animals survived to the end of the experiment.

^(1)^ – one BH showed PrPres with atypical LMW bands; this and two additional BH showed standard PrPres after sPMCAb.

^(3)^ – age-matched control animals were not inoculated, but euthanized at the age matching the age of corresponding inoculated groups.
